# MLL3 is a *de novo* cause of endocrine therapy resistance

**DOI:** 10.1002/cam4.4285

**Published:** 2021-09-28

**Authors:** Kimberly M. Stauffer, David L. Elion, Rebecca S. Cook, Thomas Stricker

**Affiliations:** ^1^ Vanderbilt University Nashville Tennessee USA; ^2^ Present address: University of California, San Francisco

**Keywords:** breast neoplasms, epigenetics, estrogen receptor alpha, genomics, MLL3 protein

## Abstract

**Background:**

Cancer resequencing studies have revealed epigenetic enzymes as common targets for recurrent mutations. The monomethyltransferase MLL3 is among the most recurrently mutated enzymes in ER+ breast cancer. The H3K4me1 marks created by MLL3 can define enhancers. In ER+ breast cancer, ERα genome‐binding sites are primarily distal enhancers. Thus, we hypothesize that mutation of *MLL3* will alter the genomic binding and transcriptional regulatory activity of ERα.

**Methods:**

We investigated the genomic consequences of knocking down *MLL3* in an MLL3/PIK3CA WT ER+ breast cancer cell line.

**Results:**

Loss of MLL3 led to a large loss of H3K4me1 across the genome, and a shift in genomic location of ERα‐binding sites, which was accompanied by a re‐organization of the breast cancer transcriptome. Gene set enrichment analyses of ERα‐binding sites in *MLL3* KD identified endocrine therapy resistance terms, and we showed that *MLL3* KD cells are resistant to tamoxifen and fulvestrant. Many differentially expressed genes are controlled by the small collection of new locations of H3K4me1 deposition and ERα binding, suggesting that loss of functional MLL3 leads to new transcriptional regulation of essential genes. Motif analysis of RNA‐seq and ChIP‐seq data highlighted SP1 as a critical transcription factor in the *MLL3* KD cells. Differentially expressed genes that display a loss of ERα binding upon MLL3 KD also harbor increased SP1 binding.

**Conclusions:**

Our data show that a decrease in functional MLL3 leads to endocrine therapy resistance. This highlights the importance of genotyping patient tumor samples for *MLL3* mutation upon initial resection, prior to deciding upon treatment plans.

## Graphical abstract

MLL3 is the 6th most commonly mutated gene in ER+ breast cancer patients, however its role in regulating the transcriptional activity of ERα in breast cancer is still unclear. Our data imply that mutations in MLL3 lend to endocrine therapy resistance through an altered genomic enhancer and ERα binding landscape resulting in an altered transcriptome, and therefore genotyping should be done prior to deciding upon course of treatment.

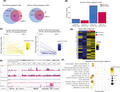

## 1 INTRODUCTION

Breast cancer is the second most commonly diagnosed cancer in American women and 75% of cases are estrogen‐receptor positive (ER+). Anti‐estrogens are the first line of therapy, however, 80% of women present with (*de novo*) or develop (acquired) endocrine therapy resistance.^1^ Disease recurrence and drug resistance are major drivers of mortality in ER+ breast cancer. While some causes of endocrine therapy resistance, such as *ESR1* mutation, *HER2* amplification, and *FGFR1*/*CCND1* amplifications are known,^2^, ^3^ ~60% of cases do not have an identified mechanism.^4^ Furthermore, only 50%–70% of ER+ patients respond to initial endocrine therapy, highlighting a need for *de novo* resistance biomarkers. Improved understanding of the mechanisms of endocrine resistance will guide therapeutic development.

ChIP‐Seq studies show tumors that respond poorly to endocrine therapy have a unique set of ERα genomic‐binding locations.^5^ Furthermore, it has been shown that ER+ breast cancer can adapt to estrogen deprivation through epigenetic reprogramming at enhancers.^6^ These patterns, therefore, suggest that genes regulating ERα binding may affect/alter endocrine therapy responsiveness. One such gene that has been shown to regulate nuclear receptor activity^7^ is *MLL3*, the sixth most frequently mutated gene in ER+ breast cancer.^8^ MLL3 primarily monomethylates H3K4 to mark enhancers. Interestingly, ERα‐binding sites regulate gene transcription largely from enhancers. In MCF7 cells, the pioneer factor FOXA1 has been shown to recruit MLL3 to demarcate enhancers for ERα.^9^ Further implicating the monomethyltransferase as an important regulator of ERα binding, MLL3 possesses LXXLL domains known to interact with nuclear hormone receptors such as ERα.^10^

Recurrent *MLL3* mutation was first identified in acute myeloid leukemia (AML), where it was determined to be a haploinsufficient tumor suppressor.^11^ Similarly, *MLL3* is recurrently mutated in ER+ breast cancer.^8^, ^12^ These mutations are predicted to be functional and therefore drivers.^13^, ^14^ Not only is *MLL3* recurrently mutated, its mutation is also associated with more aggressive disease characteristics both *in vitro* ^15^, ^16^ and *in vivo*.^17^, ^18^

Given the above observations, we predicted that mutation of *MLL3* will shift both the enhancer and ERα genomic landscape, and that this shift will affect transcriptional control by ERα and biological behavior such as endocrine resistance.

## 2 RESULTS

### 2.1 *MLL3* mutation pattern in ER+ breast cancer suggests that MLL3 is a haploinsufficient tumor suppressor

**FIGURE 1:**
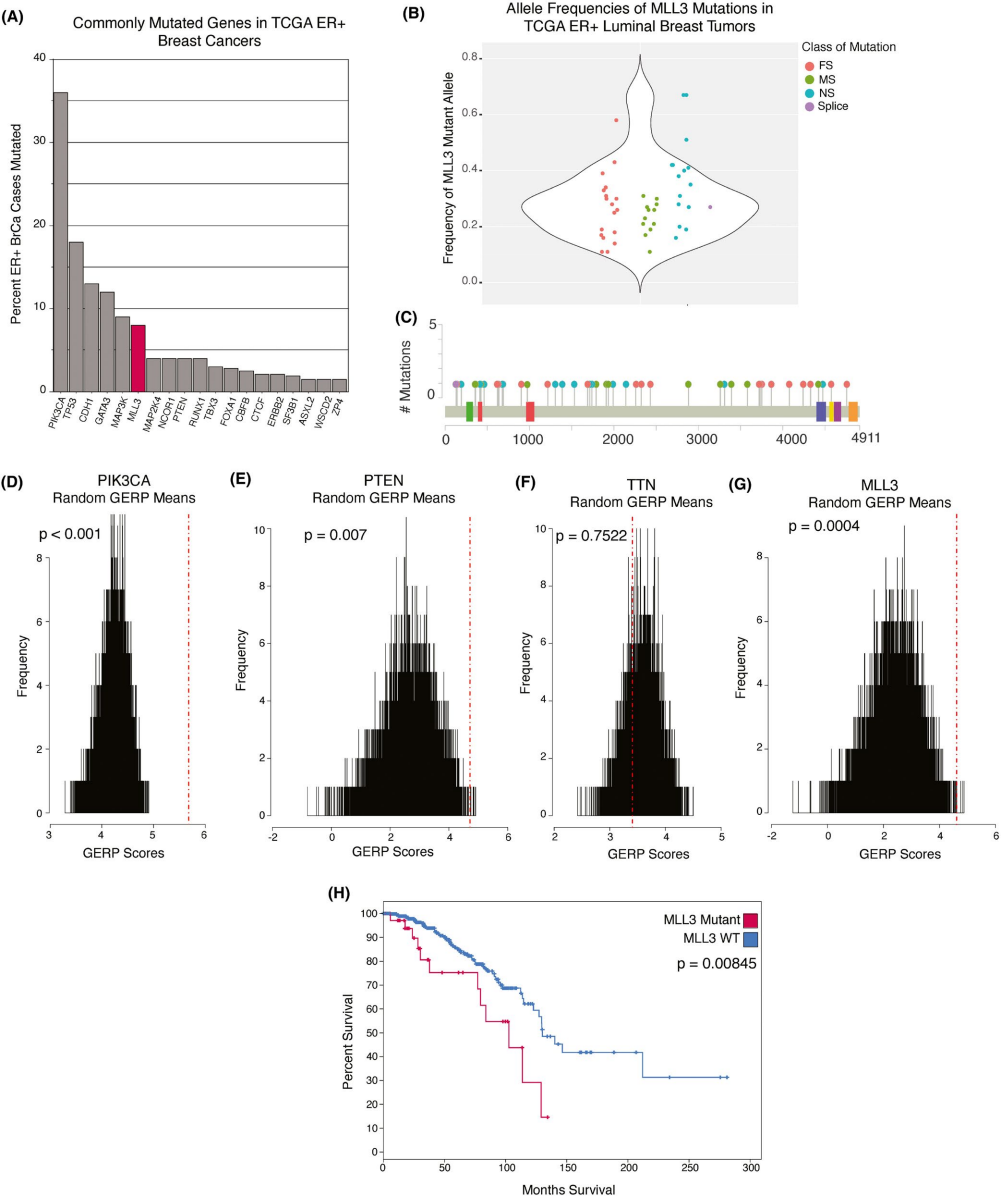
MLL3 is significantly mutated in ER+ breast cancer; its mutation confers poor outcome. (A) The most commonly mutated genes in the provisional TCGA ER+ breast cancer RNA‐seq dataset (n = 581) BrCa = breast cancer. ER+ = estrogen receptor positive. (B) Frequency of mutant MLL3 allele in TCGA ER+ luminal breast cancer cases (n = 581). FS = frameshift. MS = missense. NS = nonsense. (C) MLL3 mutation lollipop plot of luminal TCGA breast cancer cases with RNA‐seq data (n = 46 mutations). Red lollipops indicate frameshift mutations, green indicates missense mutations, blue indicate nonsense mutations, and purple indicates splice mutations. Colored boxes indicate specialty domains as follows: PHD‐like zinc‐binding (green), PHD finger (red), F/Y‐rich N‐terminus (blue), F/Y‐rich C‐terminus (yellow), catalytic SET domain (purple). (D) Histograms of (#) simulations of averages of randomly chosen GERP scores in PIK3CA (E) PTEN (F) TTN and (G) MLL3. The number of randomly chosen GERP scores matches the number of mutations in each respective gene in the TCGA luminal breast cancer cases (n = 581). Simulated averages are shown by black lines, the actual average GERP score is shown by the red dotted line. P‐values are calculated by dividing the number of simulated averages higher than the actual average GERP score by the total number of simulated averages. (H) Survival curve showing luminal cases from TCGA breast cancer cohort (n = 581) that are either mutant (red) or wildtype (blue) for MLL3. Log‐rank Test *p*‐value = 0.00845. WT = wildtype

MLL3 has been reported to be a haploinsufficient tumor suppressor in AML,^11^ and thus we hypothesized that most *MLL3* mutations in breast cancer would be heterozygous (Figure 1A).^19^, ^20^ We expect a 1:1 mutant‐to‐wildtype allele ratio in the TCGA ER+ breast cancer sample set to present as a 35:65 mutant‐to‐wildtype allele ratio for a few reasons: TCGA ER+ breast cancer samples have approximately 75% tumor purity,^21^ and copy number data from the TCGA demonstrate that no amplifications or deletions coincide with MLL3 mutations for these samples (Figure S1D). Analysis of TCGA data demonstrates that the average *MLL3* mutant allele frequency, corresponding to the percent of sequencing reads containing a mutation, is approximately 30% across the different categories of mutation (Figure 1B). This suggests that only one of two alleles is mutated, and that heterozygosity is not lost upon mutation of that one allele. This trend persists across multiple breast cancer datasets (Figure S1B), and in some of the other most frequently mutated genes in ER+breast cancer (Figure S1A). Indeed, *MAP2K4* and *TP53*, tumor suppressors associated with loss of heterozygosity,^22^, ^23^ have a higher mutant allele fraction of approximately 50%–60%. These ratios are more consistent with mutation of one allele, followed by loss of heterozygosity of the other allele in the tumor cells, given the aforementioned tumor purity.

With evidence to support that *MLL3* mutations in ER+ luminal breast cancer are heterozygous, we next considered whether the effect of the mutations would be deleterious to the function of the methyltransferase. Mutations were a mix of nonsense (16/49), frameshift (18/49,), missense (14/49), and splice (1/49) mutations spread across the length of the gene with no mutational hotspots (Figure 1C; Figure S1C). Table 1 shows that while there are no mutations within the catalytic SET domain of MLL3, there are 34 truncating mutations that occur 5’ to the SET domain. In addition, missense mutations within the PHD domains of *MLL3* have been shown to be oncogenic.^15^ Considering this information, we speculated that the 10 missense mutations outside defined regions of the protein would still lead to deleterious effects on MLL3 function.

**TABLE 1:** Domains of MLL3 and TCGA ER+ luminal breast cancer mutations

Domain Name	Function	Amino Acids	# Truncating Mutations In/Prior To	TCGA Mutations
PHD1	Putative H3/Zn binding	247–330	2	NA
PHD2	Putative H3/Zn binding	390–435	3	1ns, 2ms
PHD3	Putative H3/Zn binding	466–517	4	1ns
PHD4	Binds to H4R3me0, H4R3me2a	952–1008	10	—
PHD5	Binds to H4R3me0, H4R3me2a	1009–1055	10	—
PHD6	Binds to H4R3me0, H4R3me2a	1086–1136	10	—
LXXLL Motif	Nuclear Receptor Interacting	1408–1412	12	—
HMG‐1	DNA Binding	1655–1703	15	1fs
LXXLL Motif	Nuclear Receptor Interacting	2745–2749	23	—
LXXLL Motif	Nuclear Receptor Interacting	2918–2922	23	—
LXXLL Motif	Nuclear Receptor Interacting	3055–3059	23	—
LXXLL Motif	Nuclear Receptor Interacting	3777–3781	26	—
PHD7	Putative H3/Zn binding	4402–4506	32	1ns, 1ms
FYRN	Unknown	4550–4604	33	1fs
FYRC	Unknown	4606–4691	33	—
SET	Catalytic Domain, Methylates H3K4	4772–4893	34	—

The number of truncating mutations occurring within or prior to each domain is listed in the 4th column.

To interrogate the effect of missense mutations in the ER+ luminal TCGA cases we performed an analysis using GERP scores, an evolutionary calculation of nucleotide constraint. Genomic positions with higher scores are thought to be more deleterious if altered.^24^, ^25^ We hypothesized that the GERP scores for mutations observed in MLL3 in breast cancer would be higher, that is, more deleterious, than randomly selected missense variants, indicating that the residues mutated in TCGA samples are more conserved, and thus mutation of these conserved residues will likely be detrimental to protein function. For positive controls, we chose *PIK3CA* as an oncogene with hotspot mutations, and *PTEN* as a tumor suppressor with mutations throughout the gene.^13^ For a negative control we chose *TTN*, a known false‐positive in cancer resequencing studies. In *PIK3CA* and *PTEN*, the average GERP score of missense mutations for each gene was significantly higher, and therefore more deleterious, than the simulated GERP score averages (*PIK3CA p* < 0.0001, *PTEN p* = 0.007) (Figure 1D,E). In *TTN*, the average GERP score was within the middle of the distribution of simulated GERP averages (*p* = 0.7522) (Figure 1F). The average GERP score of missense *MLL3* mutations was on the tail of the distribution of simulated GERP averages, very similar to that of *PTEN* (*p* = 0.0004) (Figure 1G). This analysis suggests that missense mutations in *MLL3* in ER+ luminal breast cancers are deleterious to the function of the protein. Of note, a similar analysis, using the ratio of nonsynonymous to synonymous mutations in cancer also found that *MLL3* is enriched for missense mutations with evidence of selection.^13^

A Kaplan–Meier plot of TCGA breast cancer patients demonstrated that untreated ER+ breast cancer patients with *MLL3*‐mutant breast tumors have a significantly poorer overall survival than those with *MLL3*‐wildtype tumors (Figure 1H), suggesting that loss of MLL3 function contributes to poor outcomes in breast cancer patients. This trend remains true when comparing patients with *MLL3* missense mutations to patients with *MLL3*‐wildtype tumors (Figure S1E). The analyses above, along with the lack of hotspots and the number of loss‐of‐function mutations, illustrates that MLL3 is a haploinsufficient tumor suppressor in ER+ breast cancer. Thus, we decided to model *MLL3* mutation with lentiviral shRNA knockdown (KD) in the ER+ breast cancer cell line ZR751 in order to maintain some residual expression of wildtype *MLL3* (Figure S2A).

### 2.2 Knockdown of *MLL3* changes the genomic enhancer landscape

**FIGURE 2:**
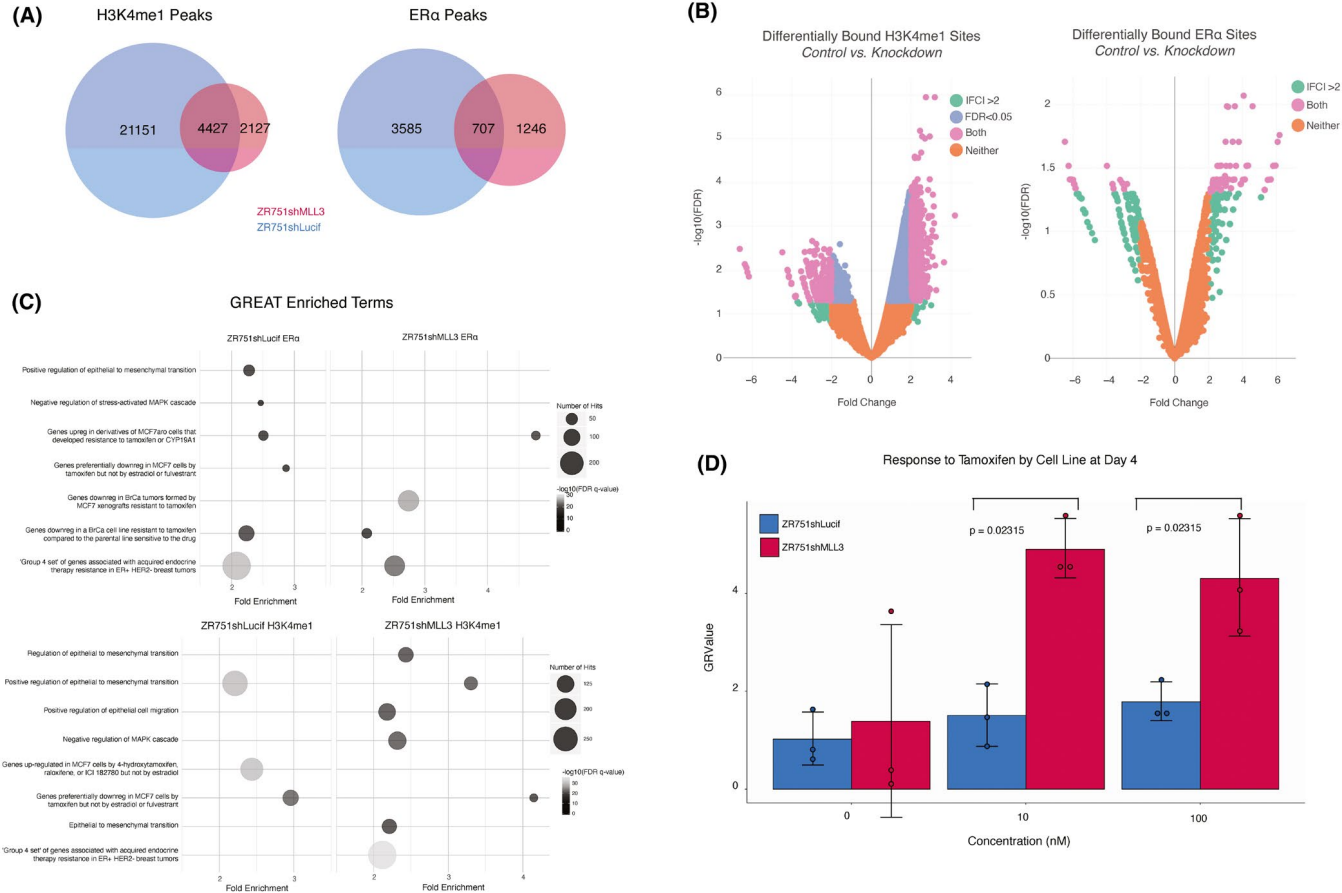
Knockdown of MLL3 leads to a reduction in H3K4me1 that correlates with a shift in ERα‐binding. (A) Venn diagrams showing either ERα or H3K4me1 peaks between merged ZR751shLucif (blue) and merge ZR751shMLL3 (red) (2 biological replicates per experiment, pooled samples with peaks chosen through IDR protocol) (B) Differentially bound H3K4me1 (left) and ERα (right) sites upon MLL3 knockdown in ZR751. Fold change and ‐log10(FDR) are plotted for the sites found by DiffBind to be differentially bound between ZR751shLucif and ZR751shMLL3. Genomic sites that have an absolute value fold change of 2 or greater are green if they do not have an FDR of less than 0.05, and pink if they do. Sites that have an FDR of less than 0.05 but do not have an absolute fold change greater than 2 are blue. Sites with an FDR of more than 0.05 and an absolute fold change of less than 2 are orange. Positive fold enrichment indicates higher amounts of binding in ZR751shLucif compared to ZR751shMLL3. FC = fold change. (C) Gene enrichment terms from GREAT for peaks that were from either ZR751shLucif or ZR751shMLL3 cells for ERα‐binding or H3K4me1 deposition. The results are displayed in matching graphs where each line on the y‐axis is a gene‐term, the x‐axis shows increasing fold enrichment, the color of the circle denotes the significance, and the size of the circle denotes the number of genes from the dataset belonging to the respective gene‐term. GREAT tool's binomial test was employed. (2 biological replicates per experiment, pooled samples with peaks chosen through IDR protocol) (D) Crystal violet assay for ZR751shLucif (blue) and ZR751shMLL3 (red) treated with Tamoxifen for 4 days. Error bars represent standard deviation. (n=3 biological replicates) (*p* = 0.02315, *p* = 0.02315, one‐sided Wilcoxon Rank Sum test of GRValues). GRValues reflect the effect of a treatment such as Tamoxifen on the growth rate of a cell population on a per‐division basis rather than on the percent viability. *GR*(*c*) = (2*(*log*2(*x*(*c*)/*x*_0_))/(*log*2(*x*(o)/*x*_0_)))−1, where *x(c)* is the number of cells in a treated well at concentration *c*, *x_0_* is the number of cells in a well at beginning of treatment, and *x(o)* is the number of cells in an untreated well

MLL3, as part of the coregulator complex ASCOM, monomethylates histone H3K4.^26^ Loss of MLL3 leads to a loss of H3K4me1 across the genome in MEF cells.^27^ We posited that loss of MLL3 function would result in a similar loss of global H3K4me1 in ER+ breast cancer. We chose to test this hypothesis in ZR751, an ER+ breast cancer cell line wildtype for *MLL3*. ChIP‐seq for H3K4me1 was performed with two biological replicates for ZR751shMLL3 and ZR751shLucif each, with inputs used as background controls. Samples were processed according to the ENCODE (phase‐3) transcription factor and histone ChIP‐seq best practices. Peak calling was accomplished with SPP^28^ and reproducibility between replicate experiments was examined to provide thresholds for optimal peak selection with the Irreproducible Discovery Rate (IDR) framework.^29^ The resulting set of peaks demonstrated a massive decrease in the number of H3K4me1 sites upon *MLL3* KD (Figure 2A). This loss is global, and comparison of H3K4me1 peaks directly shows that, on average, there is more H3K4me1 deposited at ZR751shMLL3 H3K4me1 genomic locations in control cells than in KD cells, suggesting that H3K4me1 genomic locations common to both cell lines have lower amounts of H3K4me1 in ZR751shMLL3 compared to control (Figure S3B,C). Comparison of the H3K4me1 ChIP‐seq samples with DiffBind^5^, ^30^ proved this to be true, with 97.3% (19,619/20,166, FDR < 0.05) of common H3K4me1 genomic locations having a positive fold change and therefore more H3K4me1 deposited in the control than in the KD (Figure 2B).

We reasoned that changes in the H3K4me1 enhancer landscape due to *MLL3* KD would be accompanied by genomic shifts in ERα binding. Indeed, ERα ChIP‐seq revealed a substantial shift in ERα binding upon KD of *MLL3* (Figure 2A). At genomic locations bound by ERα in ZR751shLucif, there was a greater intensity of ERα binding in ZR751shLucif cells than in ZR751shMLL3 cells, and vice versa (Figure S3B,C). Upon analysis with DiffBind we saw that indeed the differentially bound genomic locations with an FDR less than 0.05 were enriched in the ZR751shMLL3 condition if they overlapped a peak called for ZR751shMLL3, and vice versa (Figure 2B). We predicted that the altered enhancer landscape created by the loss of MLL3, comprised of major H3K4me1 loss and an altered ERα‐binding profile, would affect genes in pathways associated with cancer phenotypes. Assessment with GREAT, which assigns peaks to genes using both proximity and gene annotation categories, was used to evaluate pathway and gene signature enrichment for our ChIP‐seq data (Figure S3D). This analysis showed that, as a whole, H3K4me1 peaks in the *MLL3* KD, but not in the control, are enriched for the Creighton “group 4 set” of genes associated with acquired endocrine therapy resistance in breast tumors (Figure 2C).^31^ In the *MLL3* KD, ERα peaks are enriched for genes downregulated in breast cancers formed by MCF‐7 xenografts resistant to Tamoxifen (Figure 2C). Enrichment in these gene terms suggests that *MLL3* KD confers endocrine therapy resistance to breast cancer cells via a global loss of H3K4me1 and a shift in ERα‐binding profile. Given these results, we assessed the response of *MLL3* KD cells to endocrine therapies Tamoxifen and Fulvestrant and found that *MLL3* KD results in increased resistance to endocrine therapies (Figure 2D; Figure S2B–D).

### 2.3 Loss of functional MLL3 leads to enhanced transcription of genes associated with aggressive tumor behavior

**FIGURE 3:**
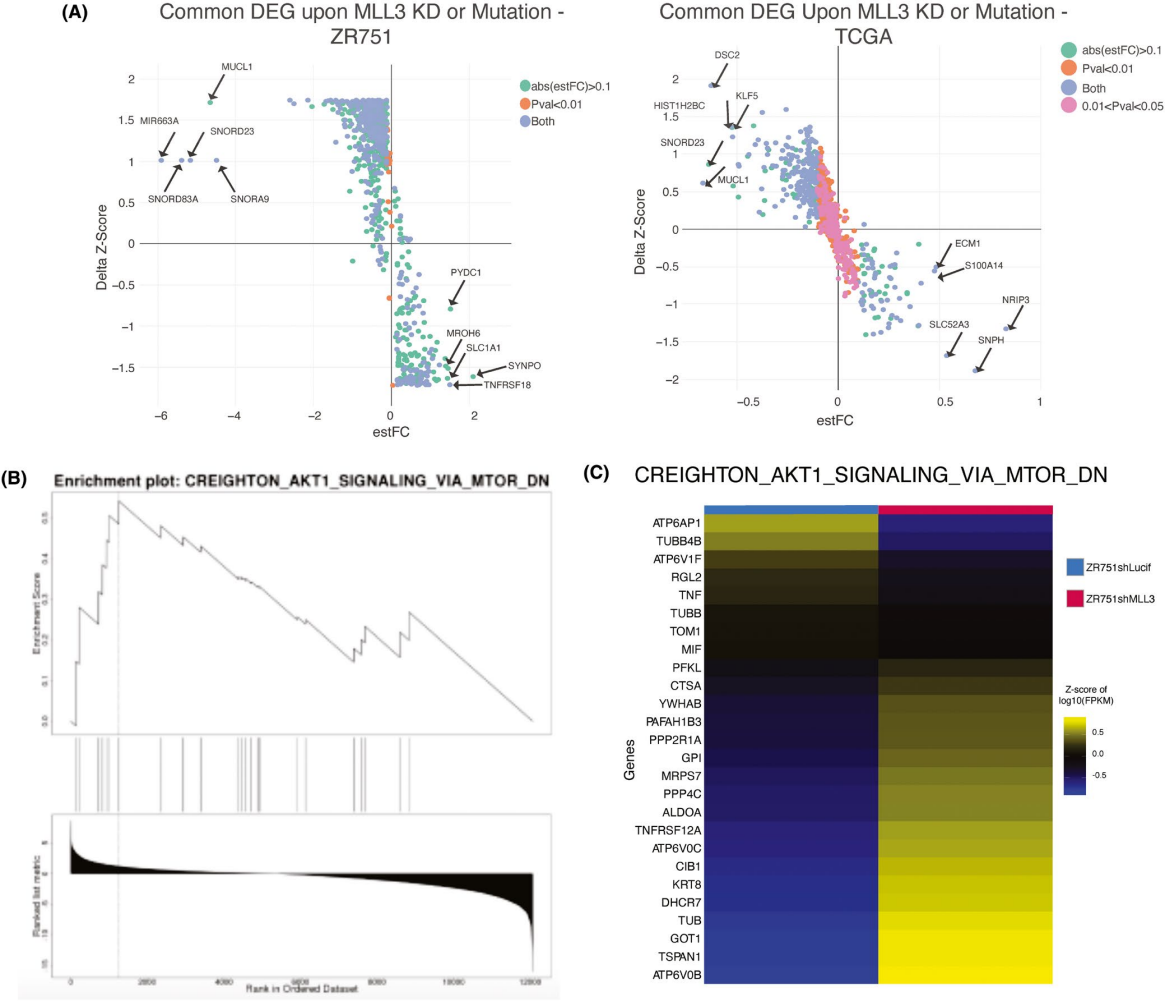
Knockdown of MLL3 and mutation of MLL3 share an MLL3‐deficiency transcriptional signature. (A) Scatterplot of the differentially expressed genes in common between ZR751 breast cancer cells upon MLL3 knockdown (left) and TCGA ER+ luminal breast cancer samples with MLL3 mutations (right). Estimated log fold change from the gene‐by‐gene linear regression model with ANOVA is plotted against the change in Z‐score between the control (ZR751shLucif on left, MLL3 wildtype samples on right) and the experimental (ZR751shMLL3 on left, MLL3 mutant samples on right). Genes with an absolute estimated log fold change greater than 0.1 are colored green if the p‐value is larger than 0.01, and blue is the p‐value is less than 0.01. Genes with a p‐value less than 0.01 and absolute estimated log fold change less than 0.1 are orange. DEG = differentially expressed genes. estFC = estimated log fold change. (B) TCGA enrichment plot for selected MSigDB term CREIGHTON_AKT1_SIGNALING_BY_MTOR_DN by WebGestalt GSEA. Normalized enrichment score 1.8757, FDR q‐value 0.026442. (C) ZR751 cell lines heatmap of Z‐scores for merged‐sample log10 normalized FPKM for genes in the CREIGHTON_AKT1_SIGNALING_BY_MTOR_DN term by WebGestalt GSEA, normalized enrichment score 2.0273, FDR q‐value 0.0026447. n = 2 biological replicates per experiments

Differential expression of RNA‐seq in ZR751shLucif and ZR751shMLL3 identified 3037 upregulated and 3518 downregulated genes upon KD of *MLL3*, q < 0.05. To determine if the same gene expression changes were occurring in clinical breast tumors with *MLL3* mutations, we utilized RNA‐seq data from TCGA ER+ luminal breast cancer patients; this analysis revealed 688 upregulated and 693 downregulated genes based on *MLL3* mutation status, q < 0.05. Comparison of the two sets of DEG from the ZR751 (q < 0.05) and TCGA (*p* < 0.05) analyses revealed a significant overlap between both upregulated (3036 ZR751, 1185 TCGA) and downregulated (3643 ZR751, 3638 TCGA) gene sets (Figures 3A, 5B; Figure S4A–D). This MLL3‐deficiency signature consisted of 208 upregulated genes (*p* = 0.0000072, Fisher's exact test) and 750 downregulated genes (750 genes, *p* = 4 × 10^−17^, Fisher's exact test).

Given the enhanced endocrine therapy resistance displayed in proliferation assays and poorer overall survival curves, we reasoned that the transcriptional program of *MLL3* KD cells would be enriched for cancer progression pathways. Webgestalt over‐representation analysis (ORA) of ZR751 DEG identified terms associated with aggressive tumor behavior due to AKT1 activation, including “genes bound by ERα and up‐regulated by estradiol in MCF7 cells expressing constitutively active AKT1” (Table S1).^32^ Webgestalt ORA of the TCGA DEG illuminated positive enrichment in *MLL3* mutants for “genes upregulated in ER+ breast cancer samples” and “KRAS‐dependency signature genes,” and negative enrichment for “genes downregulated in ER+ breast cancer samples” (Table S2). Interestingly, Gene Set Enrichment Analysis (GSEA) for both TCGA and ZR751 DEG revealed a significant positive enrichment score for “genes induced by Akt and sensitive to everolimus” (Figure 3B,C). This gene signature is correlated with an increased incidence of metastases and a shorter disease‐free survival time in several breast tumor datasets.^33^ It is worth noting that mutations in genes in the ASCOM complex, which includes MLL3, and PIK3CA pathway mutations co‐occur in breast cancer more than we would expect by chance.^34^ The mTOR pathway activation gene signature is also enriched in *MLL3* KD and mutant breast cancer samples compared to WT (Table S3). This signature is associated with poorer outcome in breast cancer compared to the pAKT pathway activation signature.^35^ These results demonstrate that canonical ERα target genes important to aggressive cancer behavior are upregulated upon loss of *MLL3*.

### 2.4 *MLL3* KD‐driven H3K4me1 loss and ERα binding shifts contribute to differential gene expression programs in breast cancer

**FIGURE 4:**
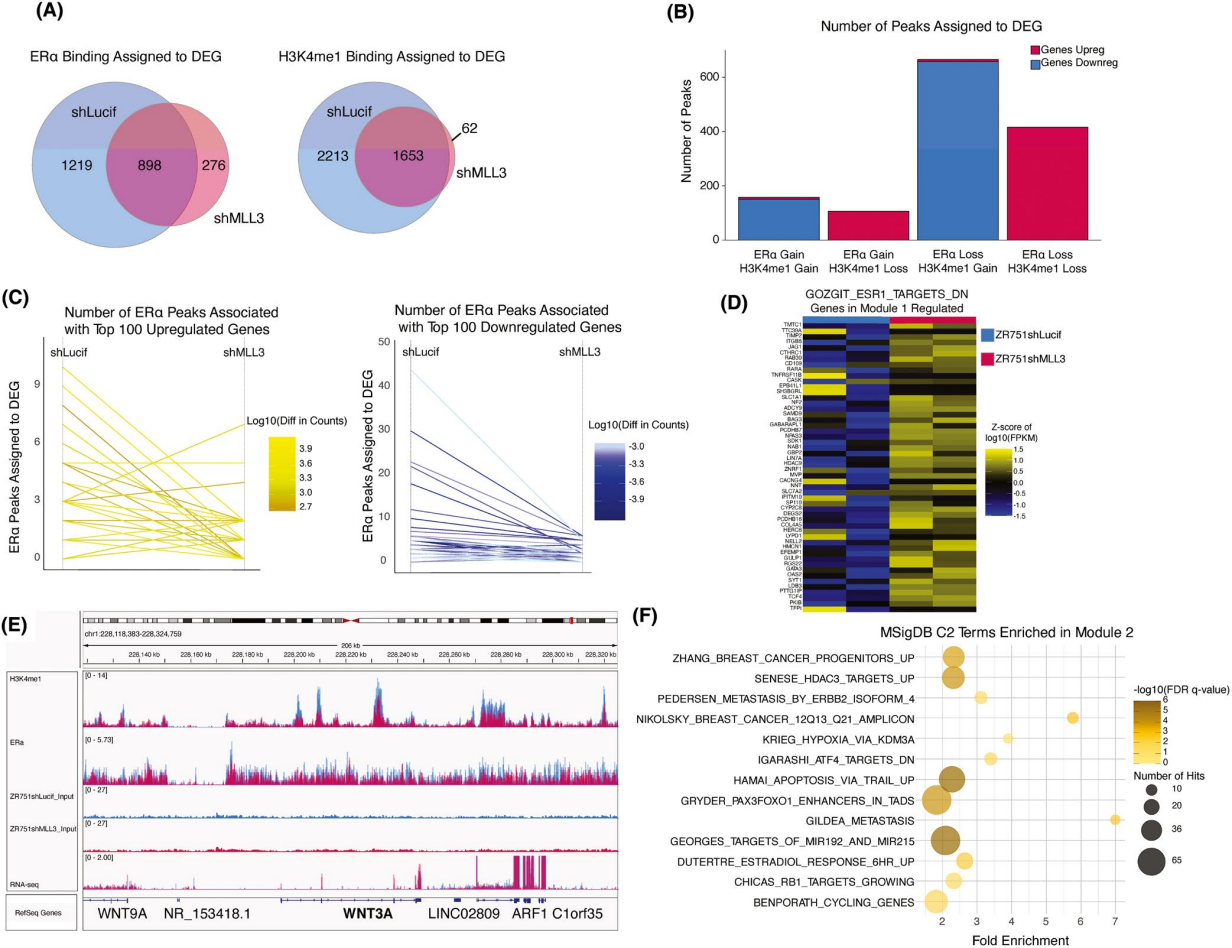
Knockdown of MLL3 in leads to a new transcriptional regulation program of ERα targets in conjunction with changes in H3K4me1 deposition. (A) Venn diagram of ERα and H3K4me1 ChIP‐seq peak assignments to differentially expressed genes (DEG) in ZR751 MLL3 KD cells. (n = 2 biological ChIP‐seq replicates per experiment) DEG = differentially expressed genes. (B) DEG upon MLL3 KD in ZR751 cells grouped into four categories based on the number of ERα and H3K4me1 ChIP‐seq peaks assigned to each gene in the control and MLL3 KD conditions. (n = 2 biological ChIP‐seq replicates per experiment) Upreg = upregulated expression. Downreg = downregulated expression. (C) Slope graph showing the difference in number of ERα ChIP‐seq peaks assigned to each DEG in ZR751s upon MLL3 KD, between the control and MLL3 KD conditions. The left graph shows the top 100 upregulated genes, and the right shows the top 100 downregulated genes. The color of each individual line represents the difference in log10‐normalized counts. (n = 2 biological ChIP‐seq replicates per experiment) (D) Heatmap of Z‐score of the log10 normalized FPKM of genes in the GOZGIT_ESR1_TARGETS_DN MSigDB term, which was significantly enriched in the Group 1 genes using WebGestalt Over Representation Analysis (ORA) (number of hits = 38, enrichment ratio = 2.1328, FDR q‐value = 0.0129) (n = 2 biological RNA‐seq replicates per experiment) (E) IGV Genome Browser snapshot of WNT3A, which belongs to Group 1 where gene expression is increased, but number of H3K4me1 and ERα ChIP‐seq peaks assigned to the gene are decreased upon MLL3 KD. (n = 2 biological ChIP‐seq replicates per experiment (F) Bubble plot showing significant MSigDB C2 terms for Group 2 genes by WebGestalt ORA. (n = 2 biological ChIP‐seq replicates per experiment)

To investigate the relationship between the changes in the genomic enhancer landscape and ERα‐binding profiles with the transcriptional changes upon KD of *MLL3*, we assigned H3K4me1 and ERα ChIP‐seq peaks to ZR751 DEG by proximity. To check the robustness of these assignments, we used a permutation‐based analysis that demonstrated our experimentally determined binding sites were closer to DEG than expected by chance (Figure S5A). H3K4me1 peaks and ERα peaks in both cell lines gave us a robustness measure of *p* = 0, and >80% of peaks were assigned for all conditions. 4179 genes out of the 6677 DEG were assigned to at least one peak (Figure 4A). We hypothesized that there would be an association between losing ERα peaks, losing H3K4me1 peaks, and decreased gene expression, and vice versa. To test this hypothesis, we next assigned each DEG to a category based on whether the number of peaks assigned to it was larger in the control or *MLL3* KD. This categorization showed a pattern in which DEG with a higher number of H3K4me1 peaks assigned to ZR751shMLL3 than ZR751shLucif tends to be downregulated rather than upregulated in ZR751shMLL3. The converse is also true (Figure 4B).

To quantify this trend we used a two‐sided Wilcoxon paired signed‐rank test with continuity correction, which confirmed that while *MLL3* KD has a sizeable effect on the number of ERα peaks assigned to DEG in both the top 100 up‐ and downregulated gene sets (*p* = 5.106 × 10^−9^, r = 0.59; *p* = 2.924 × 10^−11^, r = 0.67, respectively), a more robust effect on the number of H3K4me1 peaks assigned to DEG in the top 100 up‐ and downregulated gene sets is evident (*p* = 3.198 × 10^−15^, r = 0.853; *p* = 3.28 × 10^−12^, r = 0.871). To investigate this relationship further, the top 100 upregulated DEGs and the top 100 downregulated DEGs were dichotomized to ERα peak gain or loss and H3K4me1 peak gain or loss (Figure 4C; Figure S5B). Interestingly, the proportions of the top 100 upregulated and downregulated genes that gained ERα peaks were similar (20% of upregulated genes gained ERα peaks, and 15% percent of downregulated genes gained ERα peaks). This difference was not significant (*p*‐value = 0.4566, 2‐sample test for equality of proportions with continuity correction), suggesting that ERα peak number, per se, is not a dominating factor in determining the direction of gene expression change. However, a similar analysis for H3K4me1 peaks showed that the proportion of gained H3K4me1 peaks were vastly different (4% of the top 100 upregulated peaks, 64% of the top downregulated peaks). This difference was significant (*p*‐value <2.2 × 10^−16^, 2‐sample test for equality of proportions with continuity correction), suggesting that, interestingly, gain of H3K4me1 peaks upon loss of MLL3 is strongly associated with downregulation of gene expression.

### 2.5 Two new regulatory programs on the H3K4me1‐ERα axis drive transcriptional enrichment for *ESR1* target genes and genes associated with aggressive tumor behavior upon *MLL3* KD

To further refine our model of how loss of MLL3 enhances endocrine therapy resistance through histone mark changes and shifts in the ERα‐binding profile, we proposed that genes with similar changes in enhancer landscape, ERα binding, and direction of expression upon loss of *MLL3* would share similar biological functions. To identify genes with similar regulatory profiles and expression levels, we took an unbiased approach, grouping genes with at least one assigned H3K4me1 or ERα peak from either cell line into the four possible H3K4me1 categories: (1) H3K4me1 gain, expression upregulated (2) H3K4me1 loss, expression upregulated (3) H3K4me1 gain, expression downregulated (4) H3K4me1 loss, expression downregulated, as well as the four possible ERα categories: (1) ERα gain, expression upregulated (2) ERα loss, expression upregulated (3) ERα gain, expression downregulated (4) ERα loss, expression downregulated. Then, all 16 possible pairwise overlaps were assessed using Fisher's Exact Test with Bonferroni correction; overlaps with a significant p‐value indicate that there is a module of co‐regulated genes with that ERα and H3K4me1 status (Table 2). We tested for significant overlap between groups of DEG with either a gain or loss of assigned ERα ChIP‐seq peaks upon *MLL3* KD, and either a gain or loss of assigned H3K4me1 peaks upon *MLL3* KD. Four out of eight comparisons showed a significant overlap by one‐sided Fisher's Exact Test (*p* < 0.05) with a non‐zero Jaccard index: (1) upregulated genes with a loss in assigned H3K4me1 peaks per gene (ppg) and a loss in assigned ERα ppg upon *MLL3* KD (416 genes, *p* = 2.90 × 10–^227^), (2) upregulated genes with a loss in H3K4me1 ppg and a gain in ERα ppg upon *MLL3* KD (107 genes, *p* = 1.5 × 10^−38^), (3) downregulated genes with a gain in H3K4me1 ppg and a loss in ERα ppg upon *MLL3* KD (658 genes, *p* = 3.4 × 10^−266^), and (4) downregulated genes with a gain in H3K4me1 ppg and gain in ERα ppg upon *MLL3* KD (151 genes, 8.9 × 10^−35^). We collapsed the four groups into two modules based on the direction of effect in conjunction with H3K4me1 loss/gain (Table 2), as ERα can both drive and repress transcription of its targets. Taken together, these patterns in differential gene expression and number of associated peaks suggest that H3K4me1 peaks in WT cells that are lost after KD of *MLL3* are associated with gene upregulation, while the H3K4me1 peaks gained after KD are primarily associated with gene repression.

**TABLE 2:** Categories of regulons affected by knockdown of *MLL3*

Module	Category	H3K4me1	ERα	Direction of Effect	Overlap	P‐value	Bonferroni (α < 0.003125)	Jaccard Index	Odds Ratio
**1**	**1**	**Loss ‐ 1356**	**Loss ‐ 494**	**Upreg**	**416**	**2.90E‐227**	**Yes**	**0.3**	**29.7**
	**2**	**Loss ‐ 1356**	**Gain ‐ 159**	**Upreg**	**107**	**1.50E‐38**	**Yes**	**0.1**	**8.7**
**2**	**3**	**Gain ‐ 1150**	**Gain ‐ 333**	**Downreg**	**151**	**8.90E‐35**	**Yes**	**0.1**	**4.4**
	**4**	**Gain ‐ 1150**	**Loss ‐ 1170**	**Downreg**	**658**	**3.40E‐266**	**Yes**	**0.4**	**13.1**
3	5	Loss ‐ 0	Loss ‐ 1170	Downreg	0	1	No	0	0
4	6	Loss ‐ 0	Gain ‐ 333	Downreg	0	1	No	0	0
5	7	Gain ‐ 53	Loss ‐ 494	Upreg	8	0.039	No	0	2.2
6	8	Gain ‐ 53	Gain ‐ 159	Upreg	7	2.30E‐04	Yes	0	6.5

The table displays the organization of ZR751 differentially expressed genes (DEG) based on whether a gain or loss of associated ERα and H3K4me1 peaks were observed in the *MLL3* KD compared to the control. The background size used for the one‐sided Fisher's exact test was 6677 genes, as this was the number of DEG to which the peaks were matched. The GeneOverlap R package, by Li Shen was utilized. Categories in bold had significant overlaps. Significant categories sharing two characteristic changes were collapsed into modules.

Pathway analysis of genes belonging to the group 1 module showed significant enrichment for *ESR1* targets (52 genes, enrichment ratio 2.7435, FDR q = 6.915 × 10^−8^) (Figure 4D; Table S5). This implies that despite a decrease in regulatory H3K4me1 and ERα peaks per upregulated gene upon *MLL3* KD, *ESR1* targets are being transcribed at a higher level in *MLL3* KD cells, for example, *WNT3A* (Figure 4E; Figure S7A–F). Module 2 is enriched for several carcinogenic signatures, including “top genes down‐regulated in metastatic versus non‐metastatic bladder cancer cell lines” and “genes up‐regulated in primary melanoma, sensitive to TRAIL compared to metastatic melanoma, resistant to TRAIL” (Figure 4F; Table S6). These results suggest that apoptosis via TRAIL is being evaded in *MLL3* KD cells, and pathways involved in metastasis are being expressed at higher levels than in the control.

### 2.6 SP1 binding increases upon *MLL3* KD

**FIGURE 5:**
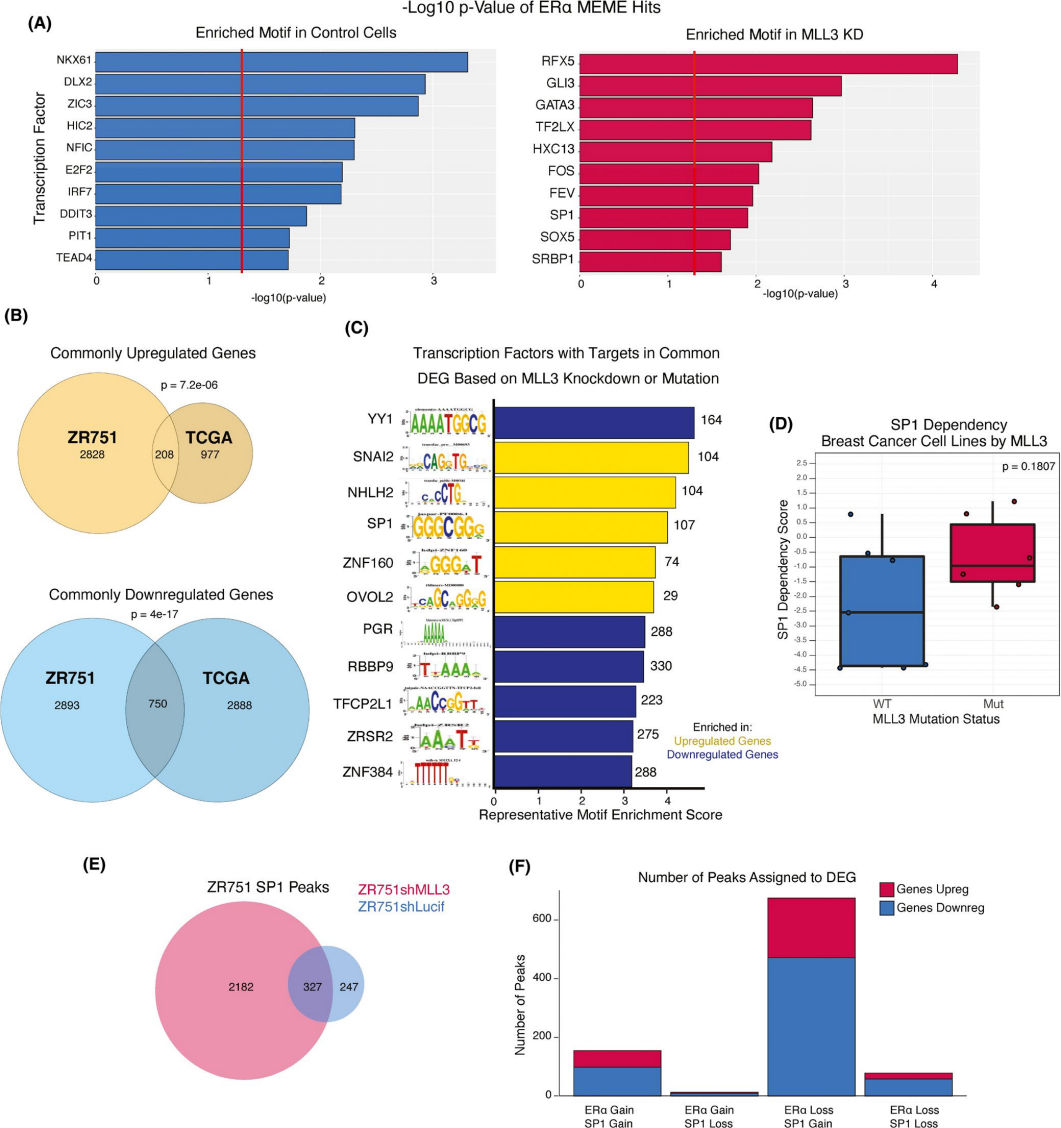
SP1 binding increases upon MLL3 KD in ER+ breast cancer cell line. (A) Representative enriched transcription factor motifs in ERα ChIP‐seq samples by MEME analysis. (n = 2 biological ChIP‐seq replicates per experiment) KD = knockdown. (B) Venn diagram of upregulated genes in the ZR751shLucif versus ZR751shMLL3 analysis as well as in the TCGA ER+ luminal breast cancer MLL3 WT versus MLL3 mutant analysis. Fisher's test, *p* = 7.2 × 10^−06^. Venn diagram of downregulated genes in the ZR751shLucif versus ZR751shMLL3 analysis as well as in the TCGA ER+luminal breast cancer MLL3 WT versus MLL3 mutant analysis. Fisher's test, *p* = 4 × 10^−17^. (n = 2 biological RNA‐seq replicates per experiment) (C) Representative enriched transcription factor motifs in the common differentially expressed genes between TCGA MLL3 WT versus mutants and ZR751 control and MLL3 KD cells, by iRegulon analysis in Cytoscape. (n = 2 biological RNA‐seq replicates per experiment) DEG = differentially expressed genes. (D) SP1 dependency scores of ER+ luminal breast cancer cell lines from the DEMETER tool where a lower score denotes a higher dependency. The center line signifies the median, box limits signify upper and lower quartiles, and whiskers signify the 1.5x interquartile range. All data points are shown as dots. Wilcoxon Rank Sum test, *p* = 0.1807 (n = 13 ER+ luminal breast cancer cell lines) (E) Venn diagram showing number of SP1 ChIP‐seq peaks in ZR751 control and MLL3 KD cell lines. (n = 2 biological ChIP‐seq replicates per experiment) (F) DEG upon MLL3 KD in ZR751 cells grouped into four categories based on the number of ERα and SP1 ChIP‐seq peaks assigned to each gene in the control and MLL3 KD conditions. (n = 2 biological ChIP‐seq replicates per experiment)

It is probable that the change in the enhancer landscape and ERα binding profile upon *MLL3* KD would be accompanied by a new milieu of transcriptional regulators responsible for aggressive behavior. To find these regulators, we interrogated motifs found in our ChIP‐seq and RNA‐seq data. We first analyzed ERα peaks that were gained upon the loss of MLL3 using MEME, which looks at the DNA sequences of the peaks to identify enrichment of binding motifs, which were then classified as belonging to transcription factors using TOMTOM. This analysis identified GATA3, FOS, and SP1 motifs enriched in ERα peaks gained after *MLL3* knockdown (Figure 5A).^36^ The iRegulon plug‐in in Cytoscape leverages both precomputed motifs and ChIP‐seq data to identify enriched transcription factor binding sites when presented with a gene list.^37^ Thus, we used genes that were differentially expressed in the same direction in both our ZR751 *MLL3* KD and in the TCGA mutant tumors to define a set of 958 differentially expressed genes as a MLL3‐deficient signature. iRegulon analysis of this gene list identified *SP1* as a candidate transcription factor for one of the top ten most‐enriched motifs for upregulated genes in the MLL3‐deficient signature (Figure 5C). Intriguingly, *SP1* was significantly upregulated in MLL3 mutants in our TCGA dataset (*p* = 2.32e‐6), although there was no statistically significant differential expression in the ZR751 cell line.

Changes in gene expression are not the only mechanism of regulation, and we hypothesized that the change in enhancer landscape might change the transcription factor milieu regardless of expression. As SP1 motifs demonstrated enrichment in both our ChIP‐seq and RNA‐seq datasets upon *MLL3* KD, we hypothesized that loss of MLL3 leads to increased activity of SP1. While the DEMETER tool for cancer‐cell line dependencies illuminated no trend toward increased or decreased dependence on SP1 for *MLL3*‐mutant ER+ breast cancer cells lines compared to those that are *MLL3*‐WT (Figure 5D),^38^ we investigated the SP1‐binding patterns in ZR751shLucif and ZR751shMLL3. ChIP‐seq for SP1 demonstrated a massive gain of 2,182 binding sites in *MLL3* KD cells (Figure 5E; Figure S6B–D). This suggests that while SP1 was not transcribed at a significantly higher rate in the *MLL3* KD, it is differentially bound to the genome depending on *MLL3* status in ZR751s.

To identify which genes SP1 regulates in control and *MLL3* KD cells, SP1 peaks were assigned to DEG in ZR751 cells using the method described for H3K4me1 and ERα. Strikingly, the largest group of DEG with both ERα and SP1 assignments are those that have an ERα peak loss and an SP1 peak gain upon *MLL3* KD. Figure 5A illustrates that upon *MLL3* KD, there is a switch from ERα to SP1 regulation of genes. Furthermore, when gene assignments between ERα and SP1 categories are compared, there is a significant overlap by one‐sided Fisher's exact test between DEG with a change in number of ERα peak assignments in *MLL3* KD cells and those with a gain in the number of assigned SP1 peaks (*p* = 1.4 × 10^−34^, Table S7; Figure S6E,F). Thus, SP1 may play a role in creating a transcriptome resistant to endocrine therapy by regulating the transcription of ERα targets that have altered ERα binding upon *MLL3* KD. In fact, 381 (nearly half of the 809 genes in Module 2) gain SP1 peaks upon MLL3 KD, while 22 of the Module 1 genes lose SP1 peaks upon MLL3 KD.

## 3 DISCUSSION

Over 40,000 women will die from breast cancer this year,^39^ and over 50% of those deaths will be due to ER+ breast cancer.^40^ ERα drives the growth of ER+breast cancers and is the target of endocrine therapy. In randomized clinical trials, endocrine therapies have effectively prevented cancer recurrence.^41^ However, approximately 20% of ER+ breast cancers will present with de novo resistance,^42^, ^43^ and many patients with early stage disease will recur after endocrine therapy.^44^ The majority of patients with metastatic ER+ breast cancer have or develop endocrine resistance, and thus both *de novo* and acquired resistance to endocrine therapy present significant hurdles to the effective treatment of breast cancer. The mechanisms underlying both *de novo* and acquired endocrine resistance remain incompletely understood, however. Somatic mutations such as *ERBB2* amplification,^45^, ^46^ ligand‐binding domain ERα mutations,^47^ and co‐amplification of FGFR1 and CCND1 have been associated with endocrine resistance, but these mechanisms do not explain even the majority of endocrine resistance.

Interestingly, both preclinical and clinical observations suggest that the majority of endocrine‐resistant tumors remain dependent on ERα. Most ER+ breast tumors retain protein expression of ERα after developing resistance.^48^, ^49^ Furthermore, about 30% of patients that develop resistance to aromatase inhibition (AI) respond to fulvestrant,^50^, ^51^ and in first‐line therapy for metastatic disease, the combination of fulvestrant and AI is superior to AI alone.^52^, ^53^ Importantly, ERα binds to different genomic locations in tumors will good versus poor outcomes, and studies show that ERα binds to different locations in endocrine‐sensitive and endocrine‐resistant cell lines,^5^ or in cell lines expressing ERα with mutations in the ligand‐binding domain (LBD). These results suggest that dysfunction of the regulatory mechanisms governing ERα genomic binding contribute to the development of endocrine‐resistant ER+ breast cancer, and we hypothesized that chromatin remodeling enzymes that can regulate the ERα genomic landscape may contribute to endocrine resistance.

MLL3, a histone monomethylase that is known to interact with nuclear hormone receptors such as ERα, is recurrently mutated in many cancers. *MLL3* is the sixth most mutated gene in ER+ breast cancer. Indeed, *MLL3* is altered in 9% of ER+ breast cancer patients in the TCGA dataset and 8.5% in the AACR GENIE dataset.^8^, ^9^ In the work above, we identify the mutation of *MLL3* as a potential common cause of endocrine resistance in ER+ breast cancer. We demonstrate that the mutation pattern of *MLL3* in breast cancer is most consistent with a haploinsufficient tumor suppressor.

Modeling loss of MLL3 function using shRNA knockdown in the ER+ PIK3CA‐wildtype breast cancer cell line ZR751, we found that knockdown of *MLL3* led to a major loss of H3K4me1 marked peaks across the genome. This loss was associated with a major shift in ERα binding, including to genes in signatures associated with endocrine resistance. Indeed, the loss of MLL3 expression increased resistance to endocrine therapy. The loss of MLL3 function was not only associated with massive changes to the H3K4me1‐marked enhancer landscape and ERα genomic‐binding sites, but also significant changes in gene expression. Assigning peaks to DEG, we were able to identify two groups of genes that were altered upon loss of MLL3. Module 1 genes demonstrate that when functional MLL3 is lost, a substantial amount of H3K4me1 marks is also lost, accompanied by a loss of ERα at those genomic locations. However, the canonical ERα target genes controlled by those lost peaks are upregulated. Module 2 genes demonstrate that a loss of functioning MLL3 results in a compensatory H3K4 methyltransferase activity that is accompanied by a change in number of regulatory ERα peaks and decreased gene expression. These two ERα‐H3K4me1‐gene modules allow breast cancer cells with a loss in functional MLL3 to increase the expression of canonical ERα targets, while also deploying transcriptional programs shown to mediate aggressive tumor behaviors.

The changes in gene expression attributed to changes in ERα regulation could be due to changes in the milieu of regulatory factors coordinating the binding of ERα to the genome. Motif analysis of both our ChIP‐seq and RNA‐seq data suggested that an SP1 transcriptional program might be activated upon inactivation of MLL3, global reduction of H3K4me1, and re‐organization of ERα genomic‐binding sites. Indeed, loss of MLL3 was associated with a massive increase in SP1 peaks. Strikingly, the largest group of differentially expressed genes with both ERα and SP1 peaks are those that have an ERα peak loss and a SP1 peak gain upon MLL3 KD. This suggests that the reorganization of the ERα‐driven transcriptome caused by loss of MLL3 results in a substantial fraction of genes being driven by SP1. Future studies will seek to identify the mechanism that unleashes SP1 in MLL3 mutant cells and its contribution to aggressive tumor behavior.

MLL3 is a member of multi‐protein epigenetic complexes, ASCOM and COMPASS.^26^, ^27^ Both ASCOM and COMPASS complexes interact with nuclear hormone receptors, including ERα. Importantly, MLL3 is not the only histone methyltransferase that can be a component of these complexes. MLL4 can also serve as the histone methyltransferase in ASCOM and COMPASS. However, each individual complex contains either MLL3 or MLL4, and the difference in their function is not well understood. Both MLL3 and MLL4 have been shown to help regulate ERα transcriptional activity, for targets such as *EZH2*, *HOX* genes, and *HOTAIR*.^27^, ^54^, ^55^, ^56^ Interestingly, *MLL4* is also recurrently mutated in many cancers, such as lung adenocarcinoma and bladder cancer,^14^ but is NOT recurrently mutated in breast cancer. It is thus possible that loss of MLL3, and its replacement with MLL4 in ASCOM complexes leads to unique histone monomethylation locations and changes in regulatory partners, like SP1, altering the transcriptional program and driving endocrine resistance. Interestingly, MLL4 has been shown to be regulated by AKT1, leading to ERα‐driven therapeutic resistance to PIK3CA inhibition. Targeted treatment of *PIK3CA*‐mutant breast cancers with anti‐PIK3CA therapy is known to lead to a compensatory increase in ER‐dependent transcription and shift in ERα genomic binding that limits therapeutic efficacy.^57^, ^58^ These changes are dependent on MLL4, suggesting that increased MLL4 function can lead to a shift in the genomic location of ERα binding that may contribute to therapeutic resistance. It has been shown that in the TCGA breast cancer dataset, increased MLL4 mRNA expression leads to shorter overall survival (*p* = 0.0398).^59^ Unsurprisingly, MLL3 KD in ZR751 cells upregulated expression of MLL4, albeit not to statistical significance, and in TCGA ER+ luminal breast cancer studies *MLL3*‐mutant cases had significantly higher expression of *MLL4* (*p* = 0.01176938, ANOVA of multivariate linear regression).

Curiously, one recent paper found that in MCF7 cells loss of MLL3 leads to decreased proliferation, decreased ERα transcriptional activity, and increased growth in estrogen‐absent media.^18^ However, MCF7 cells have a *PIK3CA* mutation, while ZR751 are wild‐type for *PIK3CA*. Thus, in MCF7 cells PIK3CA may be restraining MLL4 and ERα through activated AKT1, while MLL4 is free to activate transcription in ZR751. Future studies will focus on the interplay of MLL3, MLL4, and the PIK3CA signaling pathway. Synergies between loss of MLL3, inhibition of PIK3CA, and anti‐estrogen therapies may provide new avenues for therapy of endocrine‐resistant tumors. Furthermore, we have established that MLL3 is a haploinsufficient tumor suppressor, which suggests the possibility that loss of the remaining allele of *MLL3* could be detrimental to cancer cell survival. MLL3 is an enzyme and is thus a potential target for small molecule inhibitors. As such, subsequent studies will focus on the possibility that MLL3 and/or MLL4 may represent a therapeutic target in *MLL3*‐mutant breast cancers, as well as present a mechanism for reversal of endocrine resistance.

## 4 EXPERIMENTAL PROCEDURES

### 4.1 GERP analysis

Hg 19 base‐wise GERP scores were downloaded from http://mendel.stanford.edu/SidowLab/downloads/gerp/.^24^ To find average GERP scores for the missense mutations in each gene we used 595 TCGA ER+ luminal breast cancer cases and found the GERP score for the location of each missense mutation for the following genes: *MLL2*, *PIK3CA*, *PTEN*, and *TTN*. We calculated GERP averages for each set of missense mutations. We then selected a corresponding number of GERP scores from the entire coding sequence that would potentially lead to missense variants of each gene at random and calculated the average of those GERP scores. We repeated the random selections and average calculation 10,000 times. To get a value of significance, we divided the number of times a random selection GERP average was greater than the actual mean GERP score of our gene of interest by 10,000. Values less than 0.05 were considered significant.

### 4.2 Cell culture and antibodies

ZR751 cells (RRID CVCL_0588) were obtained from the Lannigan laboratory^60^ and grown in RPMI (Sigma Aldrich #R8758500ml) supplemented with 10% heat‐inactivated FBS (Corning™ #35016CV), 0.002% insulin (Sigma Aldrich #11376497001) and 50 IU penicillin, 50 mg/mL streptomycin (Corning™ #MT30001CI). HEK 293T cells (RRID CVCL_0063) were obtained from the Lannigan laboratory^60^ and grown in DMEM with high glucose, L‐glutamine, phenol red, but not sodium pyruvate (Sigma Aldrich D0819‐500ML), 5% FBS, 1% Pen/Strep, and 1% Sodium pyruvate (Sigma Aldrich S8636‐100ML). The cell culture incubator parameters were as follows: 37˚C, 95% relative humidity, and 5% CO_2_ concentration. The antibodies used for ChIP‐seq were anti‐Erα (Santa Cruz Biotechnology sc‐543X), anti‐H3K4me1 (Abcam ab8895), anti‐SP1 (Abcam ab13370), and sheep anti‐rabbit IgG Dynabeads M‐280 (Invitrogen™ 11203D).

### 4.3 Lentivirus‐mediated RNA‐interference (RNAi)

Oligos to use for shRNA were designed and ordered from Sigma/Genosys at the Molecular Cell Biology Core at Vanderbilt. The oligos were annealed, phosphorylated, and ligated into pSuper for transformation into DH5α cells. QIAprep Spin Miniprep Kit (Qiagen 27104) was used to isolate the vector, which was transfected into ZR751 cells and assessed by qPCR for KD. KDs that worked were then isolated with QIAprep Spin Miniprep Kit (Qiagen 27104), digested, and ligated into pLVTH^61^ (Addgene 12262) for transformation into STBL3 cells. A QIAGEN Plasmid Plus Maxi Kit (Qiagen 12963) isolated the pLVTH for transfection into HEK 293T cells, from which lentivirus was collected. The oligo sequence used to silence MLL3 was 5°‐CCGGCGCACCTTATAGTAAACAGTTCTCGAGAACTGTTTACTATAAGGTGCGTTTTT‐3°, taken from The RNAi Consortium.^62^ Negative control Luciferase shRNA Control was donated by the Lannigan laboratory.^63^ Cells were stably transduced at 100,000 cells per well in a 6‐well plate (Corning 3516) with 4 µl lentivirus, and subsequently flow sorted for GFP expression and propidium iodide (Sigma Aldrich P4864) staining after 3 days. qPCR was performed in biological triplicate to check shRNA KD 3 days after transduction. Experiments were performed in multiple, but early (<=10) passages of the stably transduced cell lines.

### 4.4 RNA‐Seq

Cells were harvested at steady state using the RNAEasy Kit (Qiagen 74104). RNA samples of 600 ng were subjected to Turbo DNAse (Thermo Scientific #AM2238) and Superscript III RT (ThermoFisher 18080093) with Random Hexamers (ThermoFisher N8080127) and dNTPs (ThermoFisher 18427088). qPCR was performed with 2 µl cDNA, 0.5 µl of 10 mM forward and reverse primers each, 10 µl SYBR Green (ThermoFisher 4364346), and 7 µl water in the Molecular Cell Biology Resource Core at Vanderbilt (BioRad CFX96 Touch Real‐Time PCR Detection System). An initial denaturation and enzyme activation step of 95°C for 3 min was performed, followed by 40 cycles of 95°C for 10 seconds to denature and 55°C for 30 seconds to anneal, and finally a melt curve. Reactions were performed in biological triplicate using SYBER green PCR Master Mix (Thermo Scientific #4344463), and results were analyzed using the delta‐delta Ct method. The average of the three biological replicate Ct values for the reference GAPDH gene was subtracted from the three individual biological replicate Ct values for the target MLL3 gene. A t‐test was performed on the resultant two groups of delta Ct values to give a p‐value of 0.0193. The Ct values ranged from 11.77 to 25.06. The qPCR was performed three times to obtain a working assay. The primers were ordered from the DNA Core at Vanderbilt from Sigma Genosys as follows: MLL3 forward, AACTCACGACCACCATCTCC, MLL3 reverse, TCTGGAGGTTTTGCATAGGG, GAPDH (control) forward, GTGAAGGTCGGAGTCAACGAPDH (control) reverse, CCCATACGACTGCAAAGACC. RNA quality was assessed in VANTAGE via Invitrogen Qubit and Agilent BioAnalyzer and samples with RIN >7 were used. RNA libraries were generated with two biological replicates of 2 μg RNA using Illumina's TruSeq Stranded Total RNA Sample Prep Kit (20020597). Libraries were sequenced at VANTAGE with PE75 to a depth of approximately 30 million reads per sample on an Illumina HiSeq3000 (Table S8). Quality of NGS data was assessed using FastQC, and adapters/low‐quality bases were trimmed from reads using fastq‐mcf from ea‐utils, with a minimum quality of 7 and a minimum length of 25. Fastq files from 595 breast invasive carcinoma samples in TCGA were downloaded from the Cancer Genomics Hub (https://browser.cghub.ucsc.edu/). Tumor classification data were obtained from the TCGA Data Portal (https://tcga‐data.nci.nih.gov/tcga/). RNA‐seq reads, both in‐house and from the TCGA, were aligned to the human genome (hg19) with Tophat (v2.0.13), quantified using cufflinks (v2.2.1), and normalized using cuffnorm (v2.2.1).^64^

### 4.5 Differential expression analysis

For ZR751 RNA‐seq, differential expression analysis was performed in Rstudio v3.6.1 using a gene‐by‐gene linear regression model with ANOVA taking MLL3 knockdown status into account. Genes with a mean expression level of log2(fpkm + 0.5) greater than 1 were kept for the analysis. A log2(fpkm + 0.5) transformation was used on the gene expression table. The sva (surrogate variable analysis) package in Bioconductor was utilized to remove batch effects.^65^ DEG were identified as those with an ANOVA FDR q‐value less than 0.05; q‐values were calculated using the qvalue package in R.

For TCGA RNA‐seq, we limited our search to breast cancer cases that were marked as ER+ in the clinical file. To decrease the variance in the control ER transcriptional activity profile, we also limited the breast cancer cases that were marked as molecular subtypes luminal A and luminal B in the clinical file. Samples that did not have information in the clinical file were discarded. Samples with an internal size factor of less than 0.35 were discarded from the analysis. Samples from men were excluded. Genes with a mean expression level of log2(fpkm + 0.5) greater than 1.5 were kept for the analysis. A transformation of log2(fpkm + 0.5) was performed on the gene expression set. The sva (surrogate variable analysis) package in Bioconductor was utilized to remove batch effects.^65^ A gene‐by‐gene linear regression model with multivariate ANOVA accounting for histological subtype, molecular subtype, and MLL3 mutation status was utilized to find differential gene expression. DEG were identified as those with an ANOVA FDR q‐value for the MLL3‐mutation status variable less than 0.05; q‐values were calculated using the qvalue package in R.

### 4.6 ChIP‐seq

ChIPs were performed for two biological replicates, for one experimental repetition. Cells were grown to 80% confluency, washed three times in ice‐cold PBS (8 g NaCl, 0.2 g KCl, 1.44 g Na_2_HP0_4_, 0.24 g KH_2_PO_4_, H_2_O up to 1 L, adjusted to pH 7.4 with HCl) and then fixed for 10 min at room temperature using 1.85% formaldehyde (50 ml cold PBS, 2.5 ml 37% formaldehyde solution Sigma Aldrich 252549), followed by quenching with 2.5 ml of 2.5 M glycine (93.8 g glycine Sigma Aldrich G7126 in 500 ml H_2_O) for 2 min at room temperature. After aspirating and washing with 50 ml cold PBS, we lysed the cells using 20 ml Farnham lysis buffer (5 mM HEPES pH 8, 85 mM KCl, 0.5% NP‐40) and 400 µl protease inhibitor cocktail (PIC, Roche 11873580001) to scrape the cells off (Corning™ 3008) into a 50 ml conical tube (Corning 352098). These tubes were spun down at 425 g for 5 min at 4°C.

Nuclei lysis buffer (50 mM Tris‐HCl pH 8, 10 mM EDTA pH 8, 1% SDS), 1X PIC, and 10 mM sodium butyrate (Sigma Aldrich B5887) were added to a concentration of 20,000,000 cells per 400 µl and resuspended until homogenous. Chromatin was sonicated using a Covaris LE220 for 35 min, then centrifuged at max speed for 10 min at 4°C to obtain supernatant. Per 0.1 ml of supernatant, we diluted with 0.9 ml ChIP dilution buffer (50 mM Tris‐HCl pH 8, 0.167 M NaCl, 1.1% Triton X‐100, 0.11% sodium deoxycholate), 0.5 ml RIPA‐150 (50 mM Tris‐HCl pH 8, 0.15 M NaCl, 1 mM EDTA pH 8, 0.1% SDS, 1% Triton X‐100, 0.1% sodium deoxycholate), 28 µl 50X PIC, and 14 µl 1 M sodium butyrate.

Anti‐ERα (3 µl/IP), anti‐H3K4me1 (1 µl/IP), and anti‐SP1 (3 µl/IP) were linked to 100 µl/IP, 60 µl/IP, and 100 µl/IP magnetic anti‐rabbit Dynabeads, respectively, with RIPA‐150 to a final volume of 500 µl for 6 h at 4°C in low‐bind tubes (Eppendorf Z666505), and then incubated with 150 µg of chromatin overnight at 4°C. Immunoprecipitants were washed with RIPA‐150 once, followed by RIPA‐500 (50 mM Tris‐HCl pH 8, 0.5 M NaCl, 1 mM EDTA pH 8, 0.1% SDS, 1% Triton X‐100, 0.1% sodium deoxycholate) twice, then RIPA‐LiCl (50 mM Tris‐HCl pH 8, 1 mM EDTA pH8, 1% Nonidet P‐40, 0.7% sodium deoxycholate, 0.5 M LiCl_2_) twice, and finally 1X TE Buffer pH 8 (10 mM Tris‐HCl pH 8, 1 mM EDTA pH 8) twice for 5 min each. Chromatin‐IPs were eluted from the beads in 200 µl freshly made Direct Elution Buffer (10mM Tris‐HCl pH 8, 0.3 M NaCl, 5 mM EDTA pH 8, 0.5% SDS), and then treated with 1 µl of 1 mg/ml RNase A (Fisher Scientific FEREN0531) at 65°C with shaking for 4 h. This was followed by 3 µl proteinase‐K (Sigma‐Aldrich 3115879001) overnight at 55°C to reverse crosslinks. DNA was purified using phenol–chloroform extraction. Samples were transferred to a spun‐down 2 ml phase lock gel tube (Qiagen 129056) and an equal volume of phenol/chloroform/isoamyl alcohol (Sigma Aldrich P3803100ML) was added and vortexed. This was spun at room temperature for 5 min at 14,000 g, and the sample was moved to a new 1.5 ml tube. One‐tenth volume of sodium acetate (Invitrogen AM9740), 1 µl glycogen (Roche 10901393001), and twice volume of 100% ethanol (Sigma Aldrich E7023500ML) were added, and the samples were incubated at −80°C for 30 mins. The sample was spun at 20,000 g for 30 min at 4°C, and the supernatant was carefully aspirated. The pellet was washed with 1 ml cold 70% ethanol, and spun at 20,000 g for 30 min at 4°C. The supernatant was aspirated, and the spin was repeated a final time. The supernatant was removed, and the pellet was allowed to dry. The pellet was then resuspended in 25 µl elution buffer (Qiagen 19086) and subsequently quantified by Qubit 2.0 Fluorometer.

Standard Illumina ChIP‐seq Library Kits (IP‐202‐1012, IP‐202‐1024) were used to build sequencing libraries for two biological replicates per condition for one experimental repetition, with inputs used as control. Libraries were sequenced at VANTAGE using an SR50 flow cell on the Illumina HiSeq3000 to a depth of approximately 20 million reads (Table S8). Quality of NGS data was assessed using FastQC v0.11.5, and adapters/low‐quality bases were trimmed from reads using fastq‐mcf from ea‐utils, with a minimum quality of 7 and a minimum length of 25. The fastq files were aligned to human genome version 19 by BWA (Burrows–Wheeler aligner Version 0.7.5a‐r405).^66^ Post‐alignment filtering was performed with Samtools 1.7^67^ and Picard 1.126 MarkDuplicates. PhantomPeakQualTools v1.2.1^68^ was used to assess ChIP‐seq enrichment quality prior to inclusion in the study, and all replicates used in this study passed. Self‐pseudoreplicates, pooled data, and pooled‐pseudoreplicates were generated and used to call peaks for the creation of peak thresholds. Peaks were called against matching input using SPP v1.15.5 according to best practices ENCODE 3 Pipeline v1.^69^ SPP uses a normalization factor is implicitly used to linearly scale the control sample for comparison with the ChIP sample; it does this by identifying a subset of background bins with a tag count exceeding Poisson density (*p* < 0.0001). Those background regions can then be normalized to the input channel. The Irreproducible Discovery Rate (IDR) framework version 2.0.3 was used to measure the reproducibility of ChIP‐seq peaks identified from replicate experiments and find thresholds based on reproducibility.^29^ All call sets used for this study met IDR benchmarks for reproducibility (Figure S3A; Figure S6A; Table S9). Final peak thresholds were chosen from this structured comparison of number of peaks called from original replicates, self‐pseudoreplicates, and pooled‐pseudoreplicates; these peak thresholds were applied to a pooled reads file composed of the two biological ChIP replicate libraries. The DiffBind package in R was utilized to find differential binding of ZR751shLucif versus ZR751shMLL3 H3K4me1, ERα, and SP1 ChIP‐seq peaks (Figure 2B; Figure S6C).

### 4.7 Peak assignment

Using Bedtools v2.26.0 we assigned each ChIP‐seq peak to the two closest DEGs rather than the closest gene in the human genome.^70^ We then removed all assignments that had a peak‐to‐gene distance greater than 1 million base pairs (bp), ranging from 16% to 26% of assignments, because most chromatin–chromatin interactions span 1 million bp or less.^71^

To determine whether our ChIP‐seq peaks are closer to our DEG than we would expect by chance, we randomly selected a matched number (6677 to equal the number of differentially expressed genes) of genes from the reference genome file to assign to our peaks, calculated distances, and then repeated this process 1000 times. A one‐sided Kolmogorov–Smirnov test between our DEG‐peak assignments and randomly chosen gene set‐peak assignments was performed for each of the 1000 repetitions, and then created a final measure of robustness by subtracting the number of *p*‐values less than 0.05 divided by 1000 from 1. Peak categories with a final measure of robustness less than 0.05 were kept.

### 4.8 Bioinformatic tools

Mutation information, survival plots, and TCGA for breast cancer samples were acquired from the National Cancer Institute Genomic Data Commons Data Portal. GRMetrics R package usage included GRfit by cell line and time point to calculate GR values. For IDR plots, peak files and an hg19 genome file were loaded into R. Parameters included half.width = NULL, overlap.ratio = 0, is.broadpeak = F, sig.value = “signal.value”. Data were processed and IDR output was generated with process.narrowpeak, compute.pair.uri, and fit.em with fix.rho2 = T as a parameter. NGS Plot heatmaps and histograms were created at the command line using ngs.plot.r with hg19 genome, with final bed files as region to plot, configuration files to plot both control and KD bam files, length from gene body of 3000 bp, ensemble as the gene database, and chipseq and protein_coding as the annotations to use. Diffbind in R utilized the DBA_EDGER analysis method with a reporting threshold of 0.1 and bUsePval = TRUE. The DBA__BLACKLIST_HG19 blacklist was applied, and a graylist.pval of 0.9 was applied afterward. A consensus peakset with a minOverlap of 0.66 and consensus of DBA_CONDITION was created and used to count reads in dba.count. These reads were normalized with dba.normalize and method = DBA_ALL_METHODS, and then contrasted with dba.contrast by condition and minMembers = 2. Analysis of differential peak enrichment was carried out using dba.analyze using DBA_ALL_METHODS. GREAT webtool version 3.0.0 was used to identify gene set enrichment analysis with ChIP‐seq data^31^ with human genome UCSC hg19 for species assembly, whole genome as background, and basal plus extension with 5.0 kb upstream, 1.0 kb downstream, and distal up to 1000 kb for associating genomic regions with genes. Curated regulatory domains were included. WebGestalt 2019 version was utilized for gene set enrichment analysis with RNA‐seq and ChIP‐seq data.^32^ RNA‐seq data were submitted to WebGestalt Gene Set Enrichment Analysis (GSEA) as rank (rnk) files sorted by ‐log10(p‐value) from the differential expression analysis in R, and the Molecular Signatures Database (MSigDB) curated gene sets of chemical and genetic perturbations (C2 CGP) database as the functional database to survey. All genes expressed in the specific dataset (ZR751 or TCGA) were used as the reference set. The minimum number of genes for a category was set at 3, and the maximum was set at 2000. P‐values from this analysis were adjusted for multiple hypothesis testing using Benjamin–Hochberg method, and the top 50 most significant terms by FDR were retrieved. Gene groups from the integration of RNA‐seq and ChIP‐seq data were submitted to WebGestalt using an Over‐Representation Analysis (ORA) using all the same parameters except for use of protein‐coding portion of the human genome as the background. The iRegulon tool v1.3 (build 2015‐02‐12) in Cytoscape software version 3.7.1 was utilized to identify enriched transcription factor motifs in DEG from RNA‐seq data^37^ with the “Predict regulators and targets” option. The species and gene nomenclature chosen was Homo sapiens, HGNC symbols, the type of search space was gene‐based, the motif collection was 10k (9713 PWMs), the track collection was ENCODE raw signals, the putative regulatory region was 20kb centered around TSS, and the motif rankings database was seven species. The Enrichment score threshold was 3.0, the ROC threshold for AUC calculation was 0.03, and the rank threshold was 5000. The minimum identity between orthologous genes for TF prediction was 0, and the maximum FDR on motif similarity was 0.001. MEME‐suite command‐line tools version 4.11.2 was used to identify enriched transcription factor motifs in ChIP‐seq data.^36^ Fasta files were used with MEME command and max dataset size of 5,000,000 letters, using the DNA alphabet, and a max number of motifs at three. Tomtom was utilized with the HOCOMOCOv11_full_HUMAN_mono_meme_format.meme database to identify known motifs within the MEME results. Dependence scores for ER+ breast cancer cell lines were acquired from the DEMETER dependence tool online at the Dependency Map (DepMap) Portal, https://depmap.org/portal/.^38^, ^41^ IGV version 2.9.4 was utilized to visualize RNA‐seq and ChIP‐seq data in the form of bigwig files, hosted at data.cyverse.org.^72^ Bigwig files were generated using command line bamCoverage program from deepTools version 3.3.1‐Python‐3.7.2 on merged bam files with the parameters bin size of 100, smoothing length of 250, normalizing using RPKM, and effective genome size using hg19.

### 4.9 Proliferation assays

Cells were plated in 96‐well plates (Fisher Scientific 07‐200‐95) with 10,000 cells per well and three biological replicates per experiment in phenol‐red free RPMI (Sigma‐Aldrich R8758500ml) with 10% heat‐inactivated charcoal‐stripped FBS (Corning™ 35016CV), 10 nM β‐estradiol (Sigma‐Aldrich E8875‐5G), 0.002% insulin (Sigma‐Aldrich 11376497001), and 50 U/ml penicillin, 50 mg/ml streptomycin (Corning™ MT30001CI), and either DMSO (Sigma‐Aldrich D8418‐100ML), Tamoxifen (Sigma‐Aldrich 579002‐5MG), or Fulvestrant (Sigma‐Aldrich I4409‐25MG). Media was switched out every four days and plates were fixed on days 4 and 8. All plates were stained with crystal violet (Sigma‐Aldrich C0775‐25G) and quantification by spectrophotometric detection at 490 nm using plate reader Molecular Devices Spectramax M3. Ten experimental replicates were performed to obtain parameters (cells per well, estradiol amount, time points) that gave consistent results. Effects were analyzed using GRmetrics version 1.10.0, one‐sided Wilcoxon Rank Sum Test, n = 3.

### 4.10 Statistical analyses

All significance level thresholds are *p* < 0.05 unless otherwise noted. For all bar‐and‐whisker plots, the center line signifies the median, box limits signify upper and lower quartiles, and whiskers signify the 1.5x interquartile range. All data points are shown as dots. For histograms and line plots, error bars represent standard deviation. Significance of survival curves (1H, S1E) was evaluated by Log‐Rank test. Quantification of gene expression (qPCR, S2A) was evaluated by a one‐tailed unpaired t‐test of the calculated delta CT values. For differential expression analyses, RNA‐seq FPKM files were log2 transformed. The R SVA package^73^ was utilized to estimate artifacts in the form of surrogate variables from the RNA‐seq data, which were then removed from the data. The cleaned data were then analyzed with a gene‐by‐gene multivariate linear regression model accounting for KD status for ZR751 data and histological subtype, intrinsic molecular subtype, and binary MLL3 mutation status for TCGA data. An ANOVA was used to evaluate the model. Estimated log expression change and Pr(>|t|) for MLL3 mutation or KD status from the linear regression and Pr(>F) for MLL3 mutation or KD status from the ANOVA were recorded for each expressed gene. Multiple hypotheses correction was achieved through the use of the qvalue R package on the ANOVA p‐values.^74^ Overlap between groups of genes was tested with the GeneOverlap R package^75^ which employs the Fisher's exact test. For proliferation assays, the R package GRMetrics was utilized to find GR values, which are the growth‐rate inhibition value of a given treatment at a given concentration. The GR values were then assessed by Wilcoxon Rank Sum Exact test, for each concentration and time point. The SP1 Dependency scores were assessed for effect by MLL3 mutation using a Wilcoxon Rank Sum exact test. The number of peaks assigned to DEG was assessed for patterns of loss or gain using both a proportions test where gain of peaks assigned to DEG in the KD condition = 1 and a loss of peaks = 0, as well as a two‐sided Wilcoxon paired signed rank test with continuity correction.

## CONFLICT OF INTEREST

The authors have no conflicts of interest to report for this article. The authors certify that they have no affiliations with or involvement in any organization or entity with any financial interest in the subject matter or materials discussed in this manuscript.

## ETHICS STATEMENT

As all data were publicly available, no ethics approval was sought.

## Supporting information

Fig S1

Fig S2

Fig S3

Fig S4

Fig S5

Fig S6

Fig S7

Table S1‐S8

Table S4

Table S9

Supplementary Material

Supplementary Material

## Data Availability

The TCGA data that support the findings of this study are openly available in the Genomic Data Commons at https://portal.gdc.cancer.gov/. The ZR751 RNA‐seq and ChIP‐seq data that support the findings of this study are available at https://www.ncbi.nlm.nih.gov/geo under series GSE163264. For codes, see online at https://github.com/staufferalexander/MLL3.
