# Supplementary figures and images for: MLL3 is a de novo cause of endocrine therapy resistance
Source: Cancer Med. 2021 Sep 28;10(21):7692–711. doi: 10.1002/cam4.4285 (PMC8559462; doi:10.1002/cam4.4285)

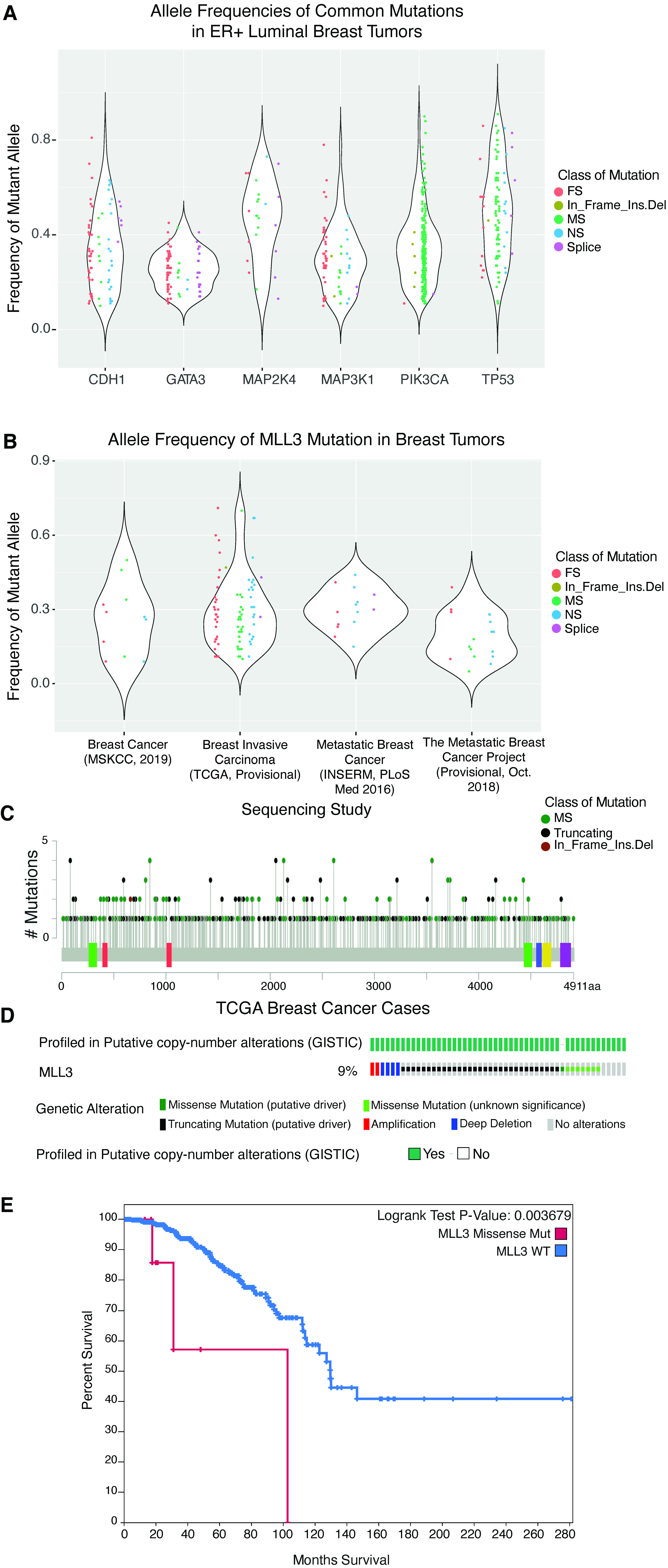

Supplement: Supplementary file 1 — Fig S1 [file CAM4-10-7692-s007.jpg]

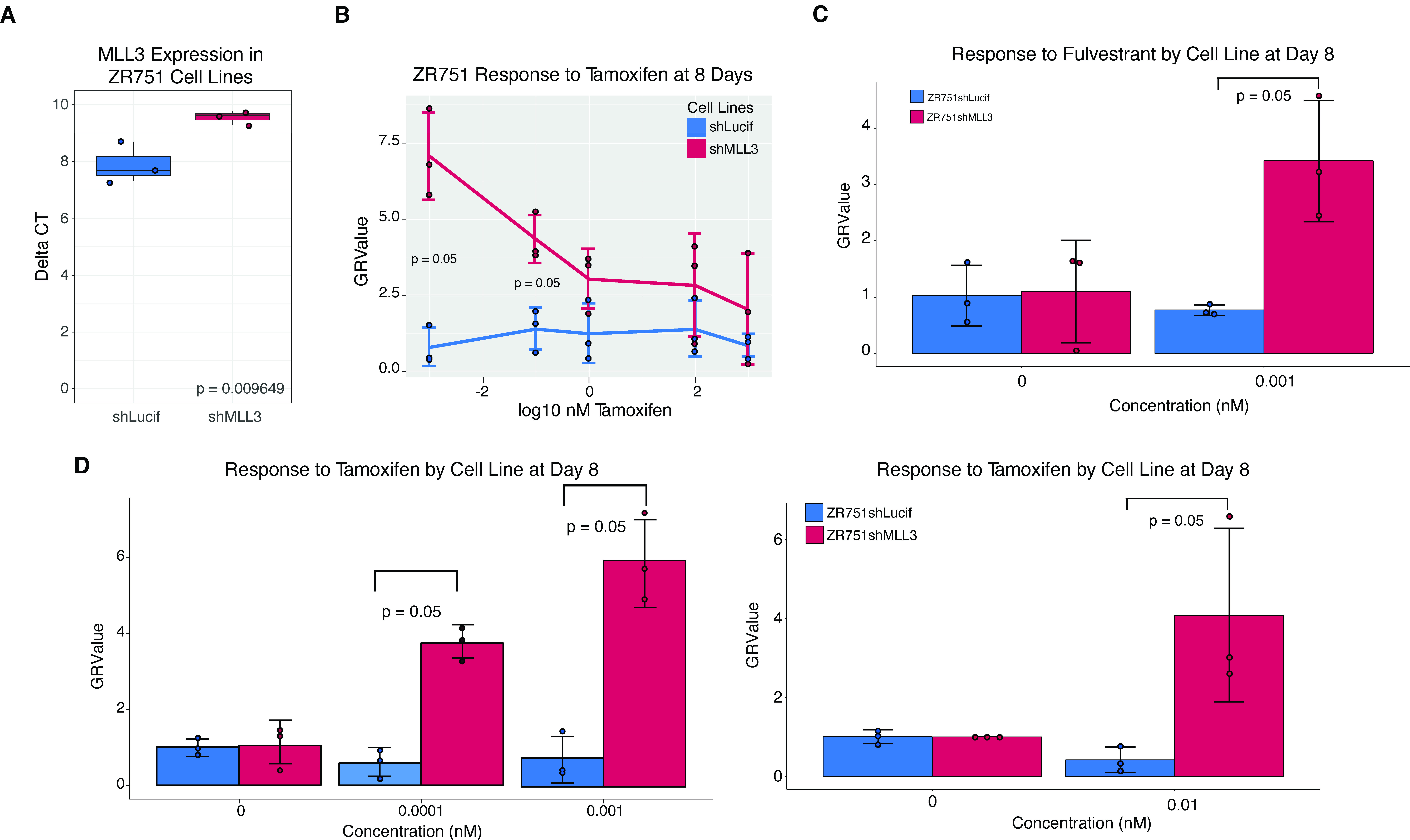

Supplement: Supplementary file 2 — Fig S2 [file CAM4-10-7692-s011.jpg]

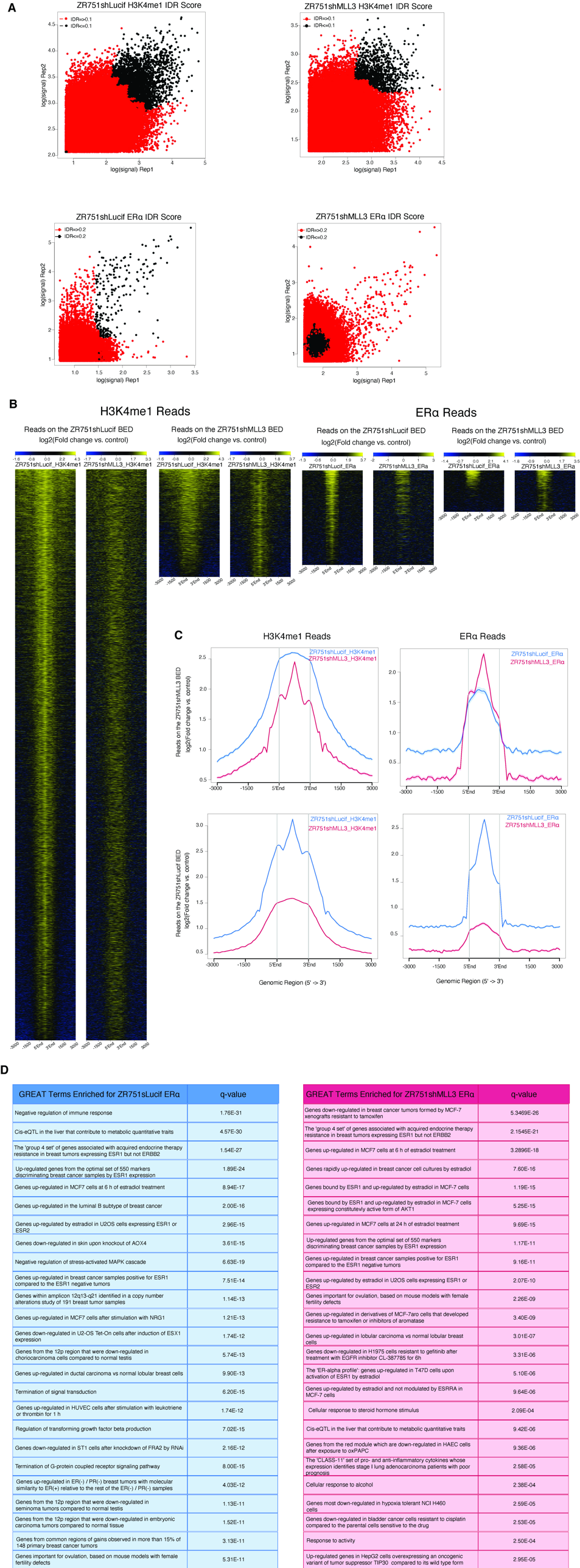

Supplement: Supplementary file 3 — Fig S3 [file CAM4-10-7692-s010.jpg]

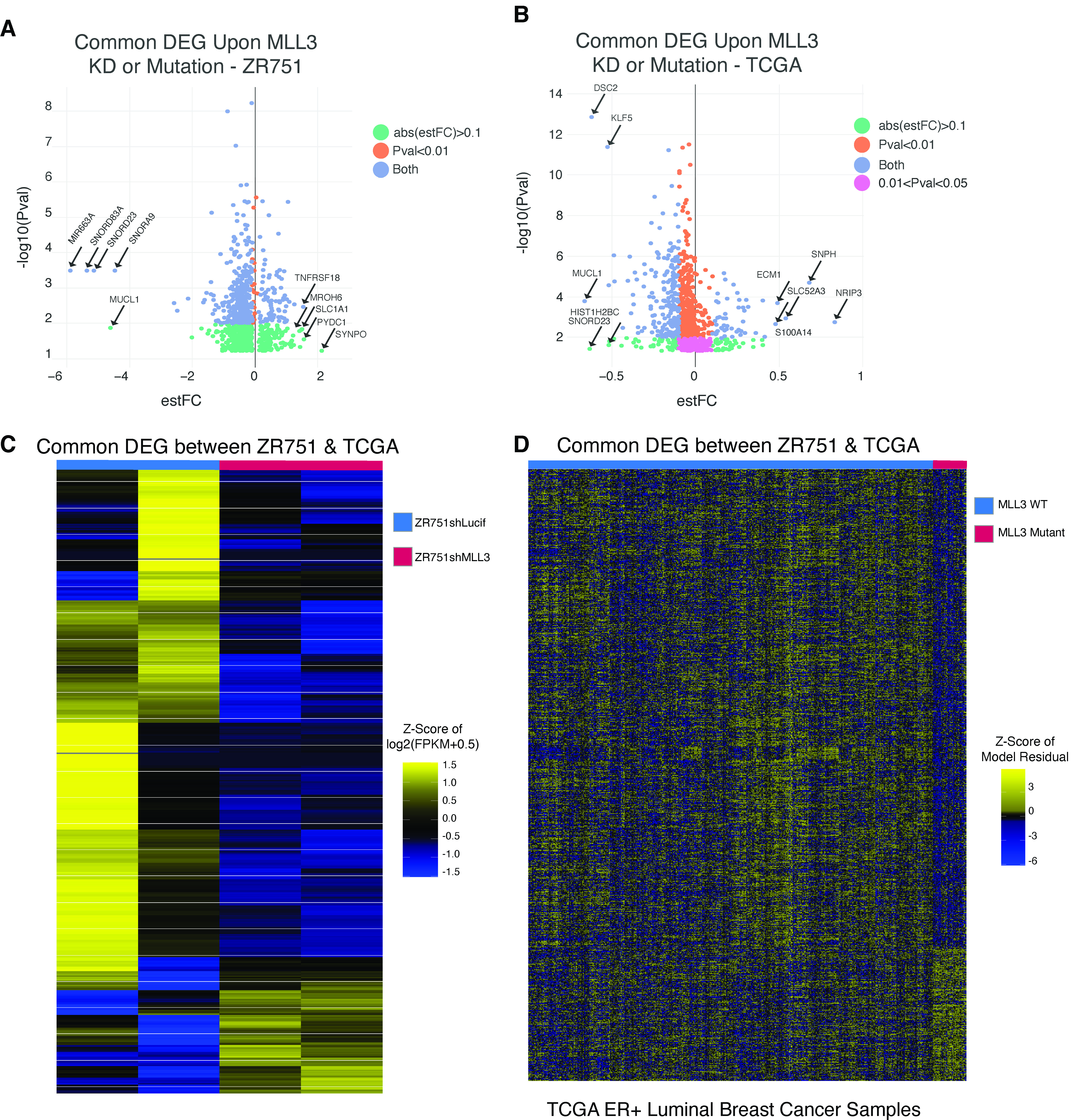

Supplement: Supplementary file 4 — Fig S4 [file CAM4-10-7692-s002.jpg]

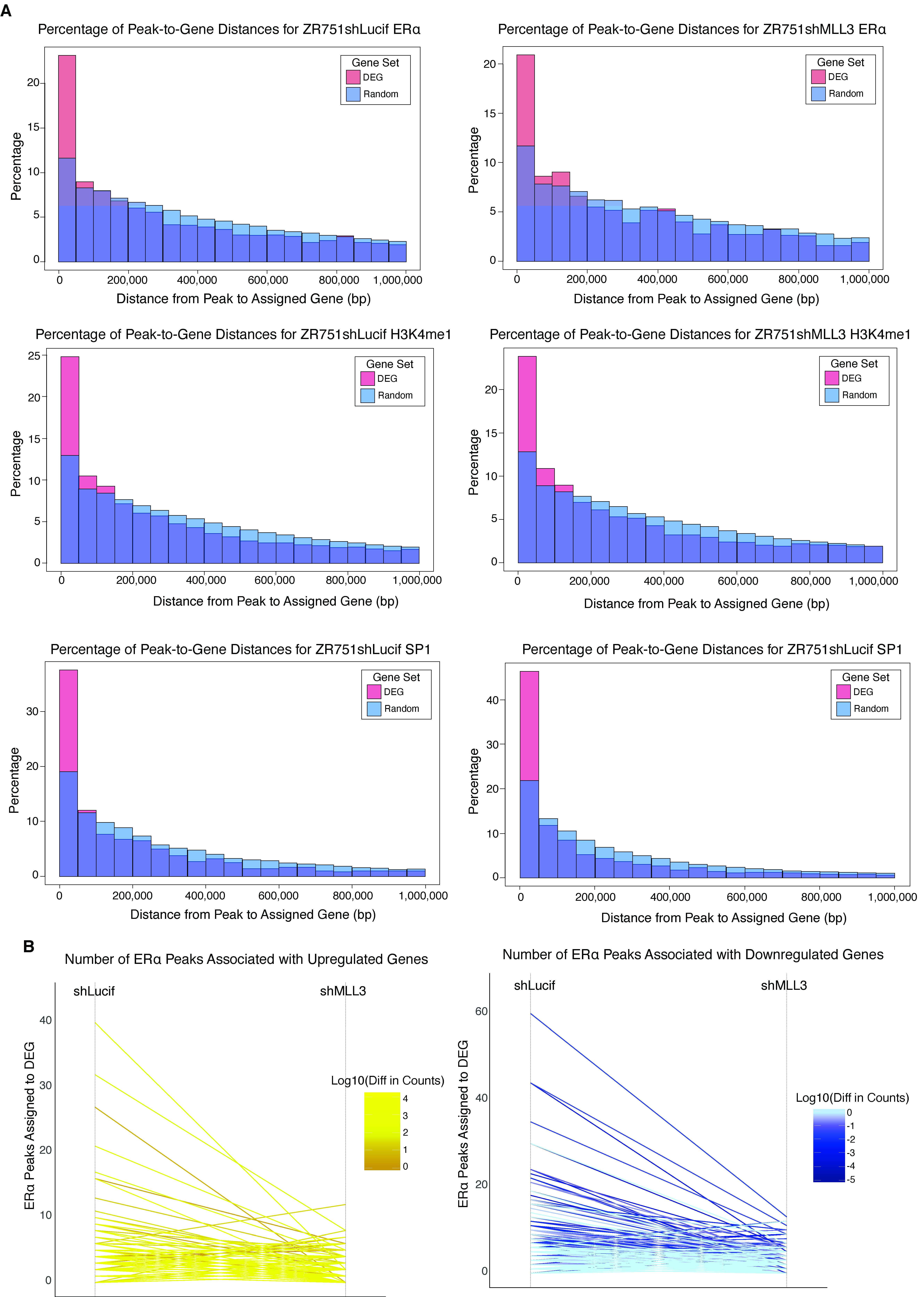

Supplement: Supplementary file 5 — Fig S5 [file CAM4-10-7692-s005.jpg]

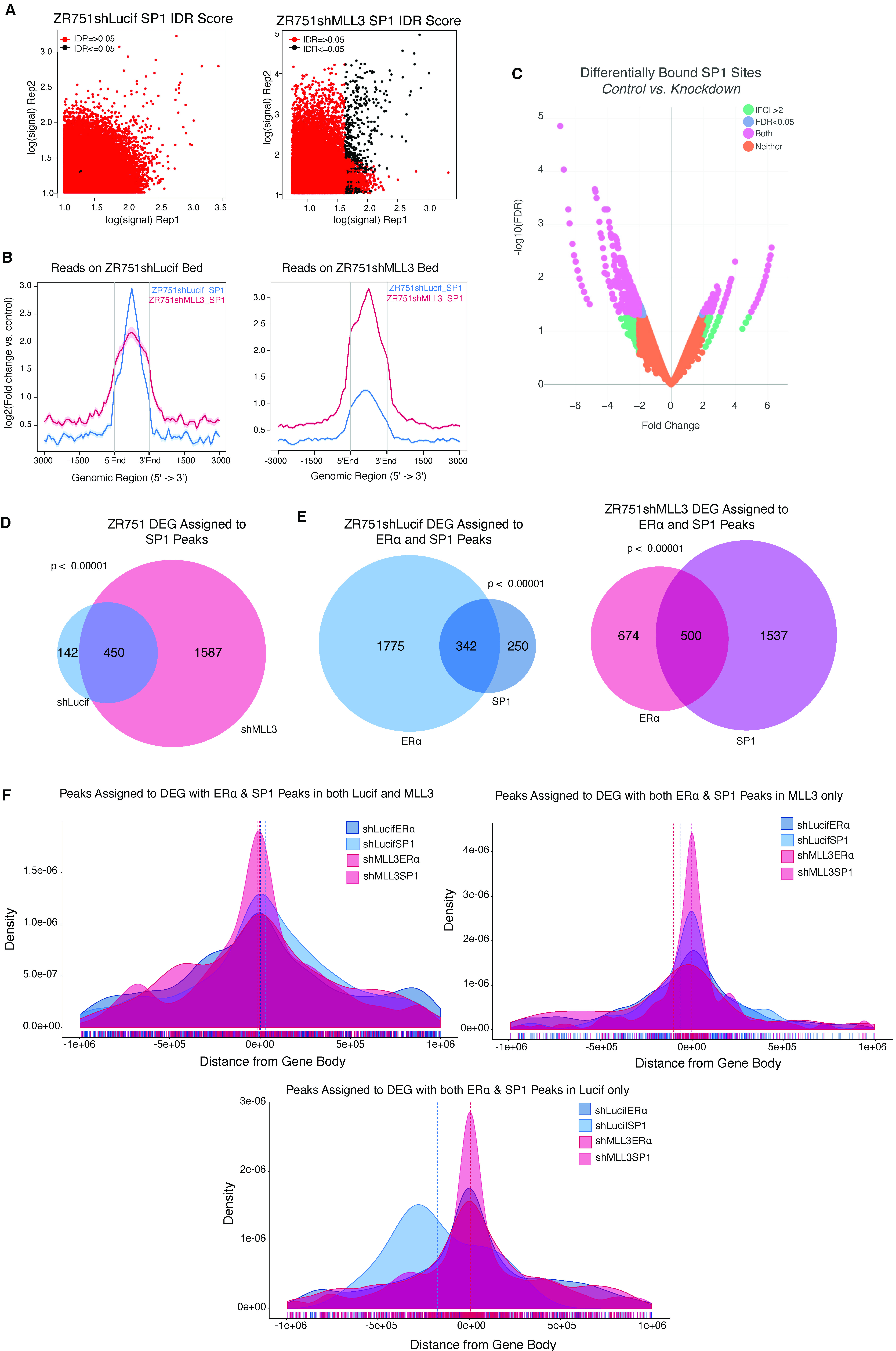

Supplement: Supplementary file 6 — Fig S6 [file CAM4-10-7692-s003.jpg]

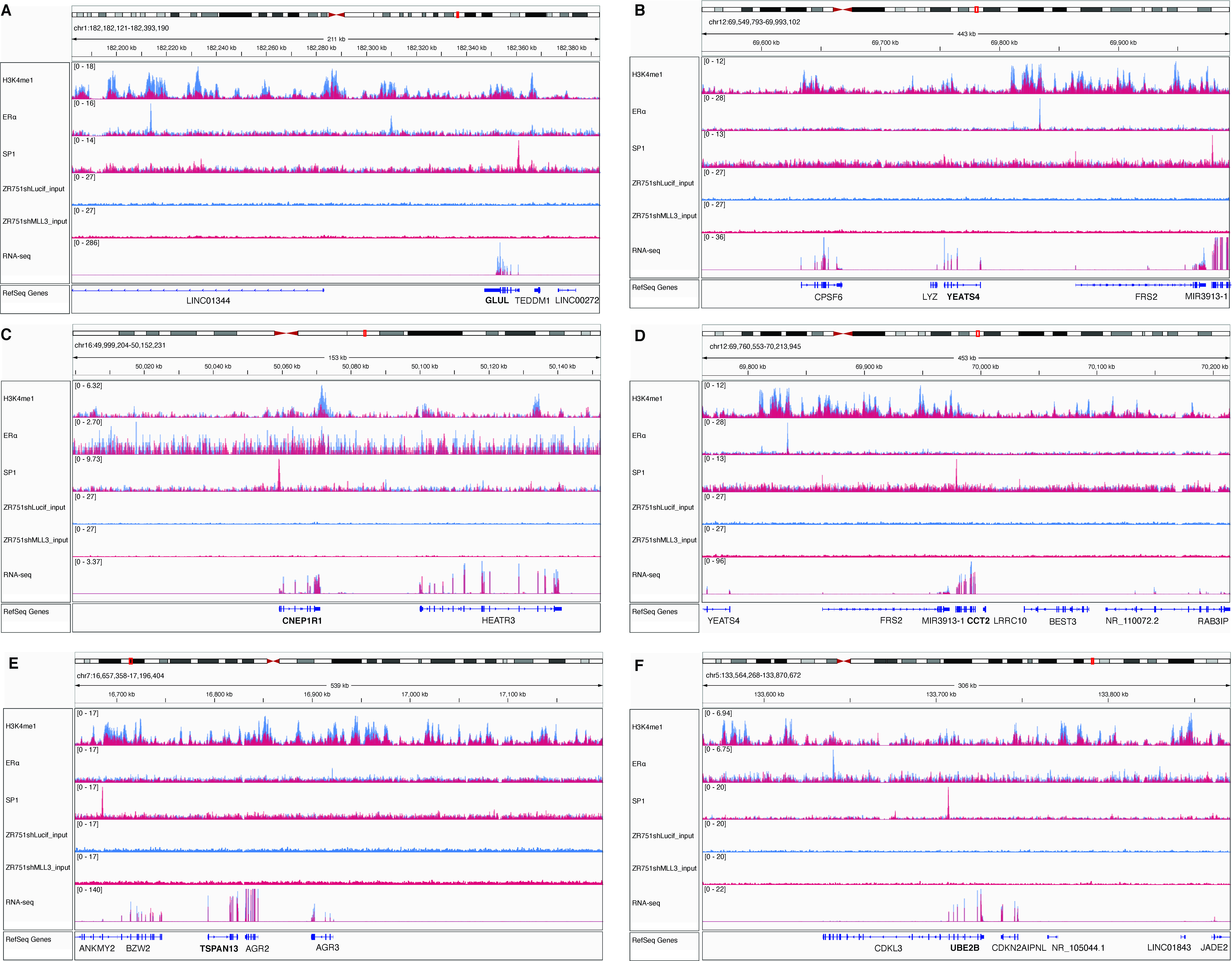

Supplement: Supplementary file 7 — Fig S7 [file CAM4-10-7692-s006.jpg]
