# Supplementary material for: MLL3 is a de novo cause of endocrine therapy resistance
Source: Cancer Med. 2021 Sep 28;10(21):7692–711. doi: 10.1002/cam4.4285 (PMC8559462; doi:10.1002/cam4.4285)
Supplement: Supplementary file 10 — Table S9 [file CAM4-10-7692-s004.docx]

| True Replicates | | Rep 1 |
| --- | --- | --- |
| shMLL3 | ERa – 0.7 | 1,736 |
|  | H3K4me1 – 0.1 | 4,541 |
|  | SP1 – 0.5 | 2,489 |
| shLucif | ERa – 0.7 | 1,483 |
|  | H3K4me1 – 0.1 | 17,242 |
|  | SP1 – 0.5 | 473 |

| Self PseudoReplicates | | Rep 1 | Rep 2 |
| --- | --- | --- | --- |
| shMLL3 | ERa - 0.7 | 3,367 | 645 |
|  | H3K4me1 – 0.25 | 3,654 | 3,730 |
|  | SP1 – 0.5 | 202 | 14,336 |
| shLucif | ERa – 0.7 | 385 | 5,305 |
|  | H3K4me1 – 0.25 | 22,345 | 7,998 |
|  | SP1 – 0.5 | 270 | 189 |

| Pooled PseudoReplicates | | Rep 1 |
| --- | --- | --- |
| shMLL3 | ERa – 0.5 | 1,511 |
|  | H3K4me1 – 0.05 | 865 |
|  | SP1 – 0.001 | 1,774 |
| shLucif | ERa – 0.5 | 3,986 |
|  | H3K4me1 – 0.05 | 24,108 |
|  | SP1 – 0.001 | 118 |

**N1 and N2 = No. of peaks passing IDR threshold by comparing self-pseudoReplicates for Rep1 and Rep2 respectively**

**Np = No. of peaks passing IDR threshold by comparing pooled pseudo-replicates**

**Nt = Best no. of peaks passing IDR threshold by comparing true replicates**

**Optimal Peak set =** **Longest of the Nt and Np peak lists**

**Rescue Ratio = max(Np,Nt) / min(Np,Nt)**
Nt and Np should be within a factor of 2 of each other

**Self-consistency Ratio = max(N1,N2) / min(N1,N2)**
N1 and N2 should be within a factor of 2 of each other

If Rescue Ratio AND self-consistency Ratio are both > 2, Flag the file for reproducibility FAIL (-1)

If Rescue Ratio OR self-consistency Ratio are > 2, Flag the file for reproducibility Borderline (0)

| Number Peaks | | Np | Nt | N1 | N2 |
| --- | --- | --- | --- | --- | --- |
| shMLL3 | ERa | 1,511 | 1,736 | 3,367 | 645 |
|  | H3K4me1 | 865 | 4,541 | 3,654 | 3,730 |
|  | SP1 | 1,774 | 2,489 | 202 | 14,336 |
| shLucif | ERa | 3,986 | 1,483 | 385 | 5,305 |
|  | H3K4me1 | 24,108 | 17,242 | 22,345 | 7,998 |
|  | SP1 | 118 | 473 | 270 | 189 |

| Ratios | | Rescue | Self-Consistency |
| --- | --- | --- | --- |
| shMLL3 | ERa - Borderline | 1.14 | 5.22 |
|  | H3K4me1 - Borderline | 5.24 | 1.02 |
|  | SP1 - Borderline | 1.40 | 75.852 |
| shLucif | ERa – Borderline | 2.68 | 13.77 |
|  | H3K4me1 - Borderline | 1.39 | 2.79 |
|  | SP1 - Borderline | 4.1 | 1.429 |
