# Supplementary material for: MLL3 is a de novo cause of endocrine therapy resistance
Source: Cancer Med. 2021 Sep 28;10(21):7692–711. doi: 10.1002/cam4.4285 (PMC8559462; doi:10.1002/cam4.4285)
Supplement: Supplementary file 11 — Supplementary Material [file CAM4-10-7692-s001.docx]

**Supplemental Figure Legends**

**Figure S1.** (**a**) Frequency of mutant MLL3 alleles in four different breast cancer sequencing studies (n =125 mutations). ER+ = estrogen receptor positive. FS = frameshift. In_Frame_Ins.Del = in frame insertion or deletion. MS = missense. NS = nonsense. (**b**) Frequency of mutant alleles in the top recurrently mutated genes in TCGA ER+ breast cancer cases (n = 554 mutations) (**c**) MLL3 mutation lollipop plot of all TCGA breast cancer cases with RNA-seq data (n= 982 breast cancer cases). Green lollipops indicate missense mutations, black indicate truncating mutations, red indicate inframe mutations. Colored boxes indicate specialty domains as follows: PHD-like zinc-binding (green), PHD finger (red), F/Y-rich N-terminus (blue), F/Y-rich C-terminus (yellow), catalytic SET domain (purple). (**d**) Snapshot of the copy number alterations and mutations in MLL3 in ER+ luminal breast cancer samples from TCGA. (n= 581 ER+ luminal breast cancer samples, only 45 samples which have copy number alterations and mutations shown) (**e**) Overall survival curve from cBioPortal comparing TCGA ER+ luminal breast cancers that has a missense mutation in MLL3 to those that are WT for MLL3. Logrank Test, p = 0.003679. (n = 581 samples) WT = wildtype.

**Figure S2.** (**a**) Delta CT values from qPCR. The center line signifies the median, box limits signify upper and lower quartiles, and whiskers signify the 1.5x interquartile range. All data points are shown as dots. (n = 3 biological replicates) (p = 0.009649, one-tailed unpaired t-test) (**b**) Crystal violet assay for ZR751shLucif (blue) and ZR751shMLL3 (red) treated with Tamoxifen for 8 days. GRValues reflect the effect of a treatment such as Tamoxifen on the growth rate of a cell population on a per-division basis rather than on the percent viability. 𝐺𝑅(𝑐)=(2*(𝑙𝑜𝑔2(𝑥(𝑐)/𝑥_0_))/(𝑙𝑜𝑔2(𝑥(o)/𝑥_0_)))−1, where *x(c)* is the number of cells in a treated well at concentration *c*, *x_0_* is the number of cells in a well at beginning of treatment, and *x(o)* is the number of cells in an untreated well. Error bars represent standard deviation. (n = 3 biological replicates) (p = 0.05, p = 0.05, one-sided Wilcoxon rank sum test) (**c**) Crystal violet assay for ZR751shLucif (blue) and ZR751shMLL3 (red) treated with Fulvestrant for 8 days. Error bars represent standard deviation. (n = 3 biological replicates) (p = 0.05, one-sided Wilcoxon rank sum test) (**d**) Crystal violet assays for ZR751shLucif (blue) and ZR751shMLL3 (red) treated with Tamoxifen for 8 days. Error bars represent standard deviation. (n=3 biological replicates each experiment) (left, p = 0.05, p= 0.05; right, p = 0.05; one-sided Wilcoxon Rank Sum test)

**Figure S3.** (**a**) IDR scatterplot of log(signal) of ZR751 H3K4me1 ChIP replicates (top) and ERα replicates (bottom). Red dots signify peaks that have an IDR score greater than the chosen threshold, 0.1 for H3K4me1 and 0.2 for ERα. Black dots signify peaks that have an IDR score of less than or equal to the chosen threshold. IDR = Irreproducibile Discovery Rate. (**b**) Heatmaps of ERα and H3K4me1 ChIP-seq reads plotted on ZR751shLucif and ZR751shMLL3 bed files of the respective ChIP-seq experiment. (n = 2 biological replicates per experiment, shown is one pooled bed file per experiment with peaks chosen through IDR protocol) (**c**) Histograms of either H3K4me1 or ERα ChIP read enrichment over control, plotted over mapped peaks from either ZR751shLucif or ZR751shMLL3. (2 biological replicates per experiment, pooled samples with peaks chosen through IDR protocol) (**d**) Chosen terms enriched in the GREAT analysis of ERα ChIP-seq experiments for ZR751shLucif and ZR751shMLL3. Q-value shown in right column of the tables (binomial test from GREAT). (n = 2 biological replicates per experiment, one pooled bed file per experiment with peaks chosen through IDR protocol).

**Supplemental Table 1 – ZR751 DEG Webgestalt**

See SupplementalTables.xlsx; gene sets explored had “estrogen” in the gene-set description or title.

**Supplemental Table 2 – TCGA DEG WebGestalt**

See SupplementalTables.xlsx; gene sets explored had “estrogen” in the gene-set description or title.

**Supplemental Table 3 – GSEA Creighton_AKT1_Signlaling_Via_MTOR_DN for ZR751 & TCGA**

See SupplementalTables.xlsx

**Figure S4.** (**a**) Scatterplot of the differentially expressed genes in common between ZR751 breast cancer cells upon MLL3 knockdown and TCGA ER+ luminal breast cancer samples with MLL3 mutations. Estimated log fold change between the control (ZR751shLucif) and the experimental (ZR751shMLL3) from the gene-by-gene linear regression model with ANOVA is plotted against the -log10(p-value). Genes with an absolute estimated log fold change greater than 0.1 are colored green if the p-value is larger than 0.01, and blue is the p-value is less than 0.01. Genes with a p-value less than 0.01 and absolute estimated log fold change less than 0.1 are orange. DEG = differentially expressed genes. estFC = estimated log fold change. (**b**) Scatterplot of the differentially expressed genes in common between ZR751 breast cancer cells upon MLL3 knockdown and TCGA ER+ luminal breast cancer samples with MLL3 mutations. Estimated log fold change between the control (TCGA ER+ luminal MLL3 wildtype breast cancer samples) and the experimental (TCGA ER+ luminal MLL3 mutant breast cancer samples) from the gene-by-gene linear regression model with ANOVA is plotted against the -log10(p-value). Genes with an absolute estimated log fold change greater than 0.1 are colored green if the p-value is larger than 0.01, and blue is the p-value is less than 0.01. Genes with a p-value less than 0.01 and absolute estimated log fold change less than 0.1 are orange. Genes with a p-value between 0.01 and 0.05 and an absolute estimated log fold change less than 0.1 are pink. (**c**) Heatmap of the differentially expressed genes in common between the ZR751shLucif vs ZR751shMLL3 analysis and the TCGA MLL3 WT vs mutant analysis, consisting of 750 downregulated genes and 208 upregulated genes. Z-scores of ZR751 expression counts of replicates are shown. ANOVA FDR q<0.05. n = 2 biological replicates per experiment. (**d**) Heatmap of the differentially expressed genes in common between the ZR751shLucif vs ZR751shMLL3 analysis and the TCGA MLL3 WT vs mutant analysis, consisting of 750 downregulated genes and 208 upregulated genes. Residuals from linear regression model *not* accounting for MLL3 mutation status used for expression values to calculate z-scores. Mutant samples are denoted by red and WT by blue. ANOVA FDR q<0.05. WT = wildtype.

**Figure S5.** (**a**) Peak-gene assignment distance for the ZR751 DEG in red, and a matched number of randomly chosen genes from hg19 for 1000 repetitions in teal. Bp = basepair. DEG = differentially expressed gene. (**b**) Slope graph showing difference in number of ERα ChIP-seq peaks assigned to each DEG in ZR751s upon MLL3 KD, between the control and MLL3 KD conditions. The left graph shows the upregulated genes and the right shows the downregulated genes. The color of each individual line represents the difference in log10-normalized counts. (n = 2 biological replicates per experiment for both RNA-seq and ChIP-seq)

**Supplemental Table 4 – Other Transcription Factors from Figure 5 in Main Body**

See SupplementalTable4_TranscriptionFactorMotifEnrichments.xlsx

**Figure S6.** (**a**) IDR scatterplot of log(signal) of ZR751 SP1 ChIP replicates. Red dots signify peaks that have an IDR score greater than the chosen threshold of 0.05 for SP1. Black dots signify peaks that have an IDR score of less than or equal to the chosen threshold. IDR = Irreproducible Discovery Rate. (**b**) Histograms of merged SP1 ChIP-seq peaks in ZR751 cells plotted on the control and MLL3 KD genomic locations. (n = 2 biological ChIP-seq replicates per experiment) (**c**) Differentially bound SP1 sites upon MLL3 knockdown in ZR751. Fold change and -log10(FDR) are plotted for the sites found by DiffBind to be differentially bound between ZR751shLucif and ZR751shMLL3. Differentially bound H3K4me1 (left) and ERα (right) sites upon MLL3 knockdown in ZR751. Fold change and -log10(FDR) are plotted for the sites found by DiffBind to be differentially bound between ZR751shLucif and ZR751shMLL3. Genomic sites that have an absolute value fold change of 2 or greater are green if they do not have an FDR of less than 0.05, and pink if they do. Sites that have an FDR of less than 0.05 but do not have an absolute fold change greater than 2 are blue. Sites with an FDR of more than 0.05 and an absolute fold change of less than 2 are orange. Positive fold enrichment indicates higher amounts of binding in ZR751shLucif compared to ZR751shMLL3. FC = fold change. (**d**) Venn diagram showing the number of ZR751 differentially expressed genes assigned to ChIP-seq SP1 peaks in ZR751shLucif and ZR751shMLL3 cells. Chi-square test of independence *X*^2^ (1, N = 6263) = 563.4442, p = < 0.00001 (n = 2 biological replicates per experiment for both RNA-seq and ChIP-seq) DEG = differentially expressed genes. (**e**) Venn diagram showing the overlap of ERα and SP1 peak-to-DEG assignments in ZR751shLucif and ZR751shMLL3 cells. Chi-square test of independence ZR751shLucif *X*^2^ (1, N = 6263) = 167.8586, p = < 0.00001, ZR751shMLL3 *X*^2^ (1, N = 6263) = 66.6957, p = < 0.00001 (n = 2 biological replicates per experiment for both RNA-seq and ChIP-seq) (**f**) Density histograms showing the distance in base pairs of the SP1 and ERα peaks in both cell lines from the gene body of the respective assigned DEGs that they regulate. (n = 2 biological replicates per experiment for both RNA-seq and ChIP-seq)

**Figure S7.** (**a**) IGV Genome Browser snapshot of GLUL, which has decreased gene expression, a higher number of H3K4me1 peaks, less ERα peaks, and more SP1 peaks assigned upon MLL3 KD. (**b**) IGV Genome Browser snapshot of YEATS4, which has decreased gene expression, a higher number of H3K4me1 peaks, less ERα peaks, and the same number of SP1 peaks assigned upon MLL3 KD. (**c**) IGV Genome Browser snapshot of CENP1R1, which has decreased gene expression, more H3K4me1 peaks, less ERα peaks, and more SP1 peaks assigned upon MLL3 KD. (**d**) IGV Genome Browser snapshot of CCT2, which has decreased gene expression, more H3K4me1 peaks, less ERα peaks, and same number of SP1 peaks assigned upon MLL3 KD. (**e**) IGV Genome Browser snapshot of TSPAN13, which has decreased gene expression, less ERα peaks, and more SP1 peaks assigned upon MLL3 KD. (**f**) IGV Genome Browser snapshot of UBE2B, which has decreased gene expression, less ERα peaks, and more SP1 peaks assigned upon MLL3 KD.

**Supplemental Table 5 – Module 1 C2_CGP WebGestalt ORA**

See SupplementalTables.xlsx

**Supplemental Table 6 – Module 2 C2_CGP WebGestalt ORA**

See SupplementalTables.xlsx

**Supplemental Table 7 – Peak to DEG Group Significance with SP1**

See SupplementalTables.xlsx

Comparisons of changes in H3K4me1, ERα, SP1, and gene expression. Groups of genes in each category were overlapped (i.e. upregulated genes with a loss in SP1 peaks vs upregulated genes with a gain in H3K4me1) and tested with Fisher’s exact test. Categories in bold had significant overlaps. Significant categories sharing two characteristic changes were collapsed into groups.

**Supplemental Table 8 – Read Counts of Sequencing Data**

See SupplementalTables.xlsx

Read Counts of RNA-seq and ChIP-seq data.

**Supplemental Table 9 – ChIP-seq IDR process**

See SuppTable9_ChIPseqnewIDR.docx

Peak thresholds from IDR process for ChIP-seq samples.
